# Modified skulls but conservative brains? The palaeoneurology and endocranial anatomy of baryonychine dinosaurs (Theropoda: Spinosauridae)

**DOI:** 10.1111/joa.13837

**Published:** 2023-02-13

**Authors:** Chris Tijani Barker, Darren Naish, Jacob Trend, Lysanne Veerle Michels, Lawrence Witmer, Ryan Ridgley, Katy Rankin, Claire E. Clarkin, Philipp Schneider, Neil J. Gostling

**Affiliations:** ^1^ Institute for Life Sciences, University of Southampton, University Road Southampton UK; ^2^ Faculty of Engineering and Physical Sciences University of Southampton, University Road Southampton UK; ^3^ School of Biological Sciences, Faculty of Environment and Life Sciences University of Southampton, University Road Southampton UK; ^4^ Department of Biomedical Sciences, Heritage College of Osteopathic Medicine, Ohio Center for Ecology and Evolutionary Studies Ohio University Athens Ohio USA; ^5^ μ‐VIS X‐ray Imaging Centre, Faculty of Engineering and Physical Sciences University of Southampton Southampton UK; ^6^ Bioengineering Science Research Group, Faculty of Engineering and Physical Sciences University of Southampton Southampton UK; ^7^ High‐Performance Vision Systems, Center for Vision, Automation and Control AIT Austrian Institute of Technology Vienna Austria

**Keywords:** Baryonychinae, endocast, palaeoneurology, sensory anatomy, Spinosauridae, Theropoda

## Abstract

The digital reconstruction of neurocranial endocasts has elucidated the gross brain structure and potential ecological attributes of many fossil taxa, including *Irritator*, a spinosaurine spinosaurid from the “mid” Cretaceous (Aptian) of Brazil. With unexceptional hearing capabilities, this taxon was inferred to integrate rapid and controlled pitch‐down movements of the head that perhaps aided in the predation of small and agile prey such as fish. However, the neuroanatomy of baryonychine spinosaurids remains to be described, and potentially informs on the condition of early spinosaurids. Using micro‐computed tomographic scanning (μCT), we reconstruct the braincase endocasts of *Baryonyx walkeri* and *Ceratosuchops inferodios* from the Wealden Supergroup (Lower Cretaceous) of England. We show that the gross endocranial morphology is similar to other non‐maniraptoriform theropods, and corroborates previous observations of overall endocranial conservatism amongst more basal theropods. Several differences of unknown taxonomic utility are noted between the pair. Baryonychine neurosensory capabilities include low‐frequency hearing and unexceptional olfaction, whilst the differing morphology of the floccular lobe tentatively suggests less developed gaze stabilisation mechanisms relative to spinosaurines. Given the morphological similarities observed with other basal tetanurans, baryonychines likely possessed comparable behavioural sophistication, suggesting that the transition from terrestrial hypercarnivorous ancestors to semi‐aquatic “generalists” during the evolution of Spinosauridae did not require substantial modification of the brain and sensory systems.

## Graphical abstract

Spinosaurids were unusual large‐bodied tetanuran theropods with semi‐aquatic ecologies. The neuroanatomy of baryonychine spinosaurids has yet to be studied, and may inform about the early evolution of the spinosaurid brain and sensory capabilities. With unexceptional hearing and olfaction, baryonychine endocasts share morphological similarities with other basal tetanurans, and our results suggest that the transition from terrestrial hypercarnivorous ancestors to semi‐aquatic “generalists” during the evolution of Spinosauridae did not require substantial modification of the brain and sensory systems.

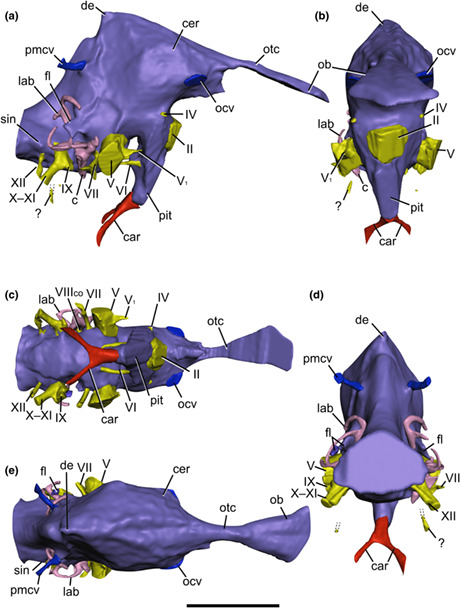

## 1 INTRODUCTION

Spinosauridae is an aberrant clade of long‐snouted, large‐bodied theropod dinosaurs, with representative taxa and key specimens known from the fossil records of Europe, Asia, Africa and South America (Allain et al., 2012; Barker et al., 2021; Charig & Milner, 1986; Dal Sasso et al., 2005; Ibrahim et al., 2014, 2020; Kellner et al., 2011; Malafaia et al., 2020; Mateus & Estraviz‐López, 2022; Sereno et al., 1998; Stromer, 1915; Sues et al., 2002). Generally limited to Lower to “mid” Cretaceous deposits (Bertin, 2010; Holtz et al., 2004), phylogenetic analyses nevertheless support a Jurassic origin for the clade (Barker et al., 2021; Carrano et al., 2012), although definitive material from this period remains unknown (Hendrickx et al., 2019; Soto et al., 2020). Spinosauridae is typically subdivided into Baryonychinae and Spinosaurinae (Arden et al., 2019; Benson, 2010; Carrano et al., 2012; Rauhut & Pol, 2019; Sereno et al., 1998), although support for this dichotomy may be weaker than previously thought (Barker et al., 2021; Evers et al., 2015; Sales & Schultz, 2017). Nevertheless, for ease of comparison, we follow the baryonychine‐spinosaurine dichotomy throughout this work.

All spinosaurids reported thus far possess atypical craniodental (and sometimes postcranial) features (Allain et al., 2012; Charig & Milner, 1997; Fabbri et al., 2022b; Ibrahim et al., 2014, 2020; Stromer, 1915), the most noteworthy of which is a long, narrow, superficially crocodile‐like rostrum. Multiple lines of evidence indicate that spinosaurids exploited divergent, semi‐aquatic ecologies relative to related lineages, which included a degree of piscivory (Amiot et al., 2009, 2010a; Charig & Milner, 1997; Hassler et al., 2018; Holtz, 1998; Hone & Faulkes, 2014; Hone & Holtz Jr, 2021; Ibrahim et al., 2014, 2020; McCurry et al., 2019; Sakamoto, 2022; Taquet, 1984). A range of terrestrial and aquatic prey were likely taken under a “generalist” or “opportunistic” foraging strategy (Bertin, 2010; Hone & Holtz Jr, 2017; Ruiz‐Omeñaca et al., 2005), with individual size (Cuff & Rayfield, 2013) or habitat (Alonso & Canudo, 2016) perhaps impacting prey availability and/or selection. Indeed, despite their apparent ties to water, spinosaurid specimens are known from several semi‐arid or arid palaeoenvironments (Amiot et al., 2010b; Ruiz‐Omeñaca et al., 2005), and direct evidence indicates that small dinosaurs and pterosaurs, in addition to fish, were consumed (Buffetaut et al., 2004; Charig & Milner, 1997). Spinosaurines may well have exhibited increased aquatic specialisation (in some respects) relative to baryonychines, but the degree to which they were adapted to water, and how this adaptation was reflected in behaviour and ecology, remains the subject of ongoing research and controversy (Arden et al., 2019; Barker et al., 2017; Fabbri et al., 2022a, 2022b; Henderson, 2018; Hone & Holtz Jr, 2019, 2021; Ibrahim et al., 2014, 2020; Myhrvold et al., 2022; Sereno et al., 2022).

Given the strong evidence for aquatic behaviour in the skeletal (and particularly cranial) anatomy of these dinosaurs, it follows that the brain and nervous system may be expected to exhibit specialisations for aquatic foraging or swimming. Schade et al. (2020) described the endocranial anatomy of the South American spinosaurine *Irritator challengeri* (SMNS 58022) from the Romualdo Formation (Lower Cretaceous: Aptian) of Brazil (Arai & Assine, 2020; Martill et al., 1996; Sues et al., 2002). *Irritator* shares similarities with the endocasts of other non‐maniraptoran theropods and possesses unexceptional hearing capabilities; more interesting is the tentative evidence from the inner ear that suggest an ability to rapidly move and tightly control ventral movements of the head that may have aided in the capture of small, agile prey such as fish (Schade et al., 2020).

Endocasts are now known for a range of theropod taxa, including early neotheropods (Paulina‐Carabajal et al., 2019; Xing et al., 2014) and ceratosaurs (Cerroni & Paulina‐Carabajal, 2019; Gianechini et al., 2021; Paulina‐Carabajal & Filippi, 2018; Paulina‐Carabajal & Succar, 2014; Sampson & Witmer, 2007; Sanders & Smith, 2005), as well as basal (i.e. non‐coelurosaur) (Eddy & Clarke, 2011; Paulina‐Carabajal & Nieto, 2019; Rogers, 1998) and coelurosaurian tetanurans (Balanoff et al., 2014; Bever et al., 2011; Brochu, 2000; King et al., 2020; Knoll & Kawabe, 2020; Kundrát, 2007; Lautenschlager et al., 2012; Witmer & Ridgely, 2009). Work on theropod endocast has also elucidated adaptational trends (Choiniere et al., 2021; Kundrát et al., 2018; Larsson et al., 2000; Zelenitsky et al., 2011) and changes in brain modularity across the theropod‐bird transition (Balanoff et al., 2016).

However, to date, no study has examined the endocranial morphology or neurosensory capabilities of baryonychine spinosaurids. Here, we fill this knowledge gap via the description and interpretation of X‐ray computed tomography (CT) scan data pertaining to *Baryonyx walkeri* and *Ceratosuchops inferodios*. Both are from Barremian strata of the Wealden Supergroup (Lower Cretaceous) of southern England, and both possess well‐preserved (albeit partially disarticulated) braincases (Barker et al., 2021; Charig & Milner, 1986, 1997). Given the temporal and phylogenetic relationship of these specimens relative to *Irritator*, the baryonychines *Baryonyx* and *Ceratosuchops* may provide some context regarding the evolution of the spinosaurid endocranium and associated neurosensory capabilities.

### 1.1 Institutional abbreviations

IGM: Geological Institute of the Mongolian Academy of Sciences, Ulaan Baatar, Mongolia; IWCMS, Isle of Wight County Museum Services, Dinosaur Isle Museum, Sandown, UK; IVPP: Institute of Vertebrate Paleontology and Paleoanthropology, Beijing, China; NHMUK, Natural History Museum, London, UK; MAU: Museo Municipal Argentino Urquiza, Rinco n de los Sauces, Neuquén, Argentina; MCF: Museo “Carmen Funes,” Plaza Huincul, Neuquén, Argentina; SMNS, Staatliches Museum für Naturkunde, Stuttgart, Germany; YPM, Yale Peabody Museum, New Haven, CT, USA.

## 2 MATERIALS AND METHODS

### 2.1 Spinosaurid material

**FIGURE 1:**
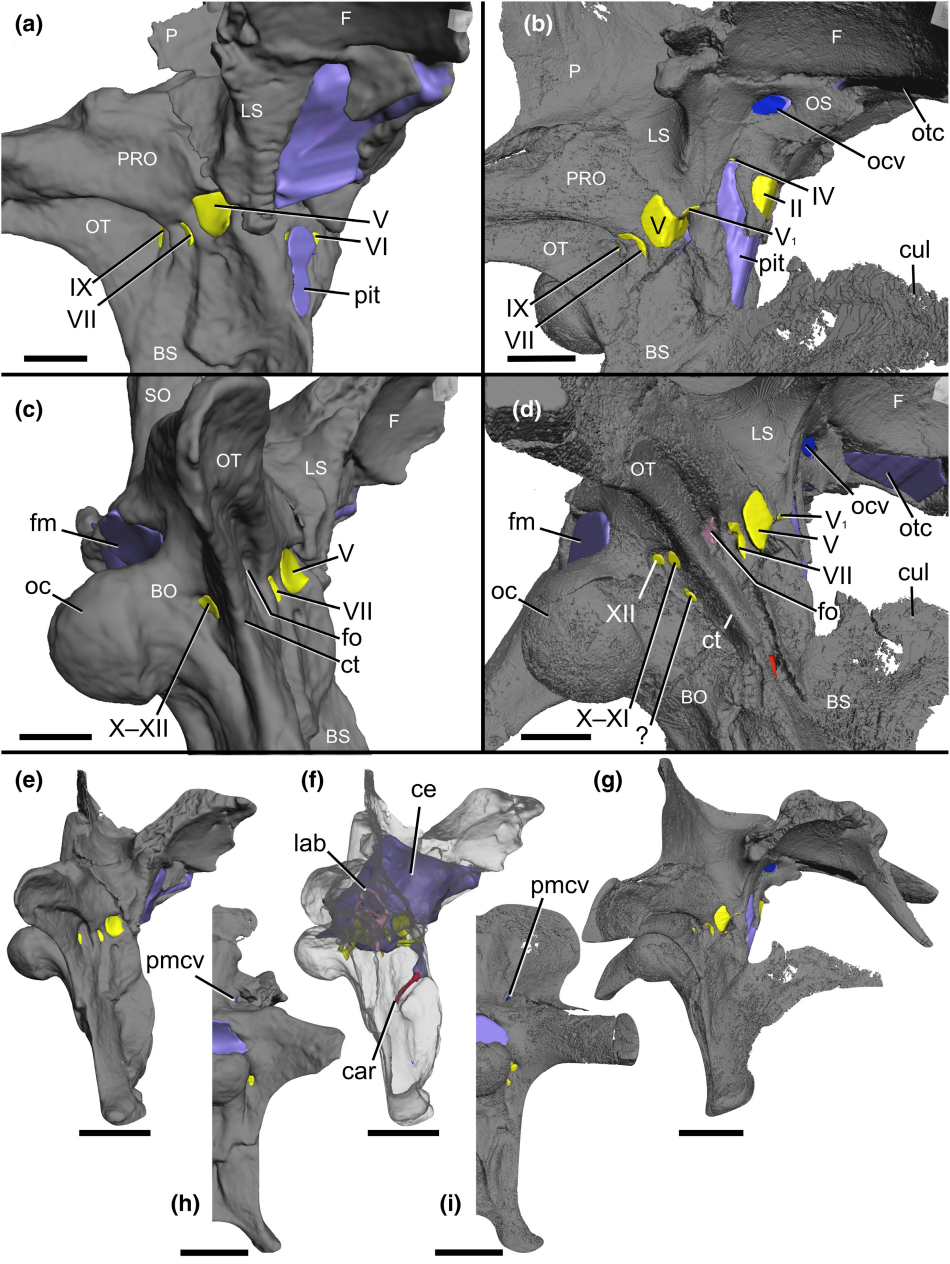
Braincases of (a, c, e, f, h) *Baryonyx walkeri* (NHMUK PV R9951) and (b, d, g, i) *Ceratosuchops inferodios* (IWCMS 2014.95.1–3), in (a, b) right anterolateral and (c, d) right posterolateral, (e–g) right lateral and (h, i) posterior (right side) views, showing the major neurovascular features (and associated foramina) and braincase anatomy. Note that in *Baryonyx*, the separate cranial nerve trunks X—XI and XII open into a common fossa lateral to the occipital condyle, which is depicted here. Abbreviations: ce, cranial endocast; cul, cultriform process; ct, crista tuberalis; BO, basioccipital; BS, basisphenoid; car, cerebral internal carotid artery canal; F, frontal; fm, foramen magnum; fo, fenestra ovalis; lab, endosseous labyrinth; LS, laterosphenoid; ocv, orbitocerebral vein; oc, occipital condyle; OS, orbitosphenoid; OT, otoccipital; otc, olfactory tract; P, parietal; pit, pituitary; pmcv, posterior middle cerebral vein canal; PRO, prootic; II, optic nerve canal; IV, trochlear nerve canal; V, trigeminal nerve canal; V_1_, ophthalmic nerve canal; VI, abducens nerve canal; VII, facial nerve canal; IX, glossopharyngeal nerve canal; X–XI, shared canal for the vagus and accessory nerves, and accompanying vessels; XII, hypoglossal nerve canal; ?, potential accessory hypoglossal nerve or venous canal. Scale bars: (a–d) 20 mm and (e–i) 50 mm.

The spinosaurid material examined first hand for this study includes the following specimens: *Baryonyx walkeri* (NHMUK PV R9951) and *Ceratosuchops inferodios* (IWCMS 2014.95.1–3) (Figure 1).

### 2.2 Terminology

Unlike that of birds or mammals, the brains of non‐avian reptiles such as *Alligator* (Hurlburt et al., 2013), turtles (Evers et al., 2019) and squamates (Allemand et al., 2022) do not typically fill the endocranial cavity. As such, a reptilian endocast represents the total soft tissues within the braincase and only provides superficial information regarding brain topography (the extent of which depending on the species and neuroanatomical regions) (Allemand et al., 2022; Hopson, 1979; Hu et al., 2021). As such, we follow previous works by referring to the digital casts of the space within the braincase as “endocasts”. Similarly, the segmented labyrinths do not truly represent the membranous or osseous features of the inner ear, and we also follow previous authors in adopting “endosseous” throughout when referring to the reconstructed structure (Witmer et al., 2008). Other segmented structures (e.g. neurovascular canals, pituitary fossae) are referred to as if they were the structures themselves.

### 2.3 CT scanning and endocast generation

#### 2.3.1 Data archiving

The data used in this study were archived on Morphosource (https://www.morphosource.org/projects/000491312). Data pertaining to *Baryonyx walkeri* and *Ceratosuchops inferodios* can be respectively found under project IDs 000491194 (https://www.morphosource.org/projects/000491194) and 000491949 (https://www.morphosource.org/projects/000491949).

#### 2.3.2 *Baryonyx walkeri* (NHMUK PV R9951)

##### CT scanning

The preserved braincase consists of three fragments (i.e. a large posterior basicranial fragment, a smaller skull roof fragment with portions of the frontals, parietals and left laterosphenoid, and the left otoccipital with its paroccipital process) that were scanned at OhioHealth O'Bleness Hospital in Athens, Ohio, USA, on a General Electric LightSpeed Ultra at 140 kV and 200–300 mA, with voxel sizes of 432 × 432 × 625 μm and using Extended Hounsfield and bow‐tie filtration.

##### Volume reconstruction and image processing

Scan data were reconstructed using a bone algorithm and were exported as a DICOM image stack, which were then imported into Windows‐based workstations running Amira‐Avizo (Thermo Fisher Scientific) for analysis and segmentation. To generate 3D PDFs, 3D models were exported as OBJ files and imported into SimLab 3D Composer (Amman, Jordan).

##### Braincase rearticulation

**FIGURE 2:**
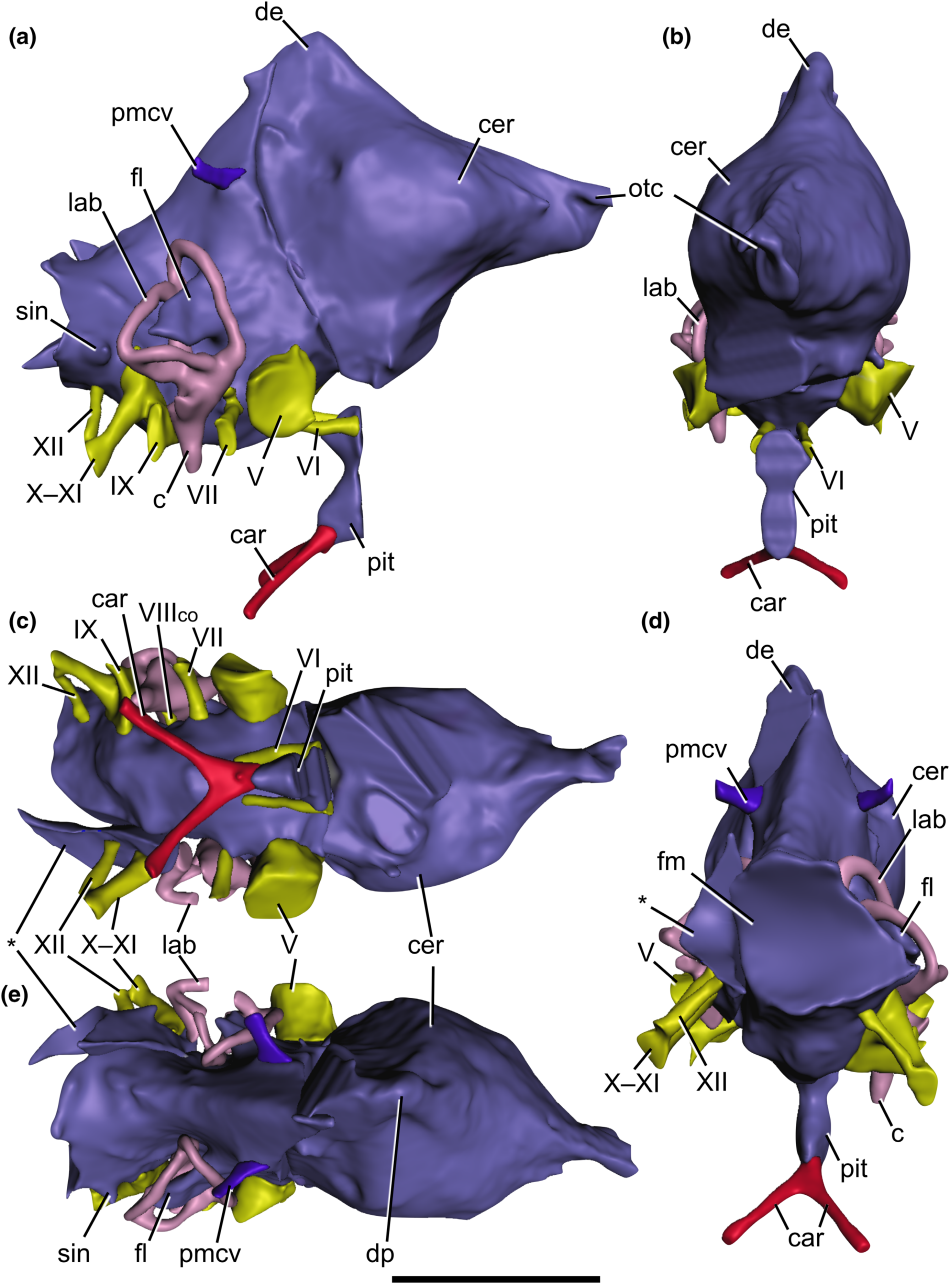
Cranial endocast of *Baryonyx walkeri* (NHMUK PV R9951), reconstructed from CT scans, in (a) right lateral, (b) anterior, (c) ventral, (d) posterior and (e) dorsal views. Vascular structures and endosseous labyrinth also depicted. Abbreviations: c, cochlea; car, cerebral internal carotid artery canal; cer, cerebral hemisphere; de, dorsal expansion; fl, floccular lobe; lab, endosseous labyrinth; otc, olfactory tract; pit, pituitary; pmcv, posterior middle cerebral vein canal; sin, blind dural venous sinus of the hindbrain; V, trigeminal nerve canal; VI, abducens nerve canal; VII, facial nerve canal, VIII_co_, cochlear ramus of the vestibulocochlear nerve; IX, glossopharyngeal nerve canal; X–XI, shared canal for the vagus, and accessory nerves, and accompanying vessels; XII, hypoglossal nerve canal. Asterisk (*) marks the disarticulated left otoccipital portion of the endocast. Scale bar: 50 mm.

The three preserved braincase fragments show some amount of plastic deformation and, although close, do not fit together perfectly. Different models were generated based on different optimization criteria: (1) best overall fit considering both bony structure and enclosed soft tissues (e.g. semicircular canals); (2) best fit optimizing bony structure, especially the otoccipital fragment; and (3) best fit optimizing the structure of the labyrinth, which is the most consistently symmetrical aspect of endocranial anatomy (Cerio & Witmer, 2019). The differences between the resulting models are slight, and Figures 1 and 2 presents the result of the second optimization criterion above. It is also worth noting that portions of the bony braincase overlying the left cerebral region were not preserved (or were lost during collection or preparation) but the underlying cerebral endocast is preserved in matrix as a natural endocast.

#### 2.3.3 *Ceratosuchops inferodios* (IWCMS 2014.95.1–3)

##### CT scanning

The three braincase fragments were scanned at the μ‐VIS X‐Ray Imaging Centre at the University of Southampton (UK), using a custom 225/450 kVp Hutch dual source walk‐in micro‐focus CT system (Nikon Metrology, UK). Peak voltage and current were set at 300 kVp and 250 μA respectively. A total of 3142 projections were collected over a 360° rotation, averaging 16 frames per projection with 250 ms exposure time per frame.

##### Volume reconstruction and image processing

Projection data were reconstructed as 32‐bit float raw volumes with an isotropic voxel size using filtered back‐projection algorithms implemented in CT Pro 3D and CT Agent software (v. XT 2.2 SP10, Nikon Metrology, UK). Voxel dimensions were 103.3 μm^3^ for IWCMS 2014.95.1, 89.3 μm^3^ for IWCMS 2014.95.2, and 109.7 μm^3^ for IWCMS 2014.95.3. These were converted to 8‐bit (raw) volume files to reduce computational load.

To improve contrast, the raw image files of the basicranium fragment (IWCMS 2014.95.3) were manipulated in FIJI (Schindelin et al., 2012) using the “sharpen” filter and background subtract function. Additional sharpening of the *Ceratosuchops* braincase material was conducted within Object Research Systems (ORS) Dragonfly (v. 2022.1, build 1249) via the *Unsharp* filter (*Workflow* > *Image Filtering* > *Operations* > *Sharpening*) using a factor of 4–9 (standard deviation = 1), depending on the specimen/region of interest (ROI).

##### Braincase rearticulation and segmentation

A landmark‐based rearticulation process in 3D Slicer (v. 4.11.20210226) (Fedorov et al., 2012) was used to reconstruct the braincase, whilst segmentation of the endocranial features was achieved using ORS Dragonfly (Object Research Systems (ORS) Inc, Montreal, Canada; v. 2021.3 and 2022.1). Final endocast generation employed Autodesk Meshmixer. The methodological details are expanded upon in the Supplementary information.

### 2.4 Measurements

Morphological measurements were taken digitally using the measuring tools in Autodesk Meshmixer, ORS Dragonfly and FIJI (Schindelin et al., 2012).

### 2.5 Reptile encephalisation quotient

The reptile encephalisation quotient (REQ) (Hurlburt, 1996) is a measure of relative brain size and gross cognitive capacities. We generated an estimated REQ based on the brain and body mass of the more skeletally complete *Baryonyx* type specimen. Body mass estimates are unknown for *Ceratosuchops*, but both taxa are of similar proportions where their known anatomy overlaps (Barker et al. (2021); see also Table 1), and the REQ estimated for *Baryonyx* likely approximates that of *Ceratosuchops*. The equation is as follows:

$$\mathrm{REQ}=\frac{M_{\mathrm{Br}}}{0.0155\times M_{{\mathrm{Bd}}^{0.553}}}$$

where M_Br_ is brain mass (in grams, excluding the olfactory tract and bulbs) and M_Bd_ is body mass (in grams). Following Hurlburt et al. (2013), we assumed the brain occupied 37% or 50% of the endocast volume. As the relative density of brain tissues nears unity, brain volume and mass are interchangeable (Hurlburt et al., 2013). Endocast volume was calculated in Meshmixer (*Analysis* > *Stability*).

**TABLE 1:** Metric data for the baryonychine endocasts.

	*Baryonyx*	*Ceratosuchops*
Preserved anteroposterior length (measured from the anterodorsal‐most tip to the dorsal margin of the foramen magnum)	128.4*	173.5
Anteroposterior length (excluding olfactory tract)	128.4	122.2
Endocranial volume (excluding neurovascular features)	150.487*	185.603
Mediolateral width across cerebral hemispheres	47.5	47.9
Cephalic flexure	63°	78°
Pontine flexure	55°	66°

*Note*: Linear measurements in millimetres (mm). Volume data in cubic centimetres (cm^3^). Asterisk (*) denotes incomplete data due to preservation.

A mass estimate for *Baryonyx* was generated based on the equation of Campione et al. (2014) implemented in Benson et al. (2018):

$${\text{mass}}_{\text{biped}}=\frac{10^{\left(2.749\times \log_{10}\left(\mathrm{FC}\times 2^{0.5}\right)-1.104\right)}}{1000}$$

where FC is minimum femoral circumference (in millimetres). Inputting the available femoral data for *Baryonyx* (minimum preserved circumference of the incomplete right shaft: 350 mm; S. Maidment, pers. comms., 2022) produced a mass estimate of 2011 kg. However, the measured *Baryonyx* femur is not only incomplete but also damaged (Charig & Milner, 1997), which affected the ability to collect reliable data (S. Maidment, pers. comms., 2022). Nevertheless, this estimate is comparable to the 1981 kg calculated by Therrien and Henderson (2007) using least‐squares regressions involving skull length, and is used herein pending the discovery of better preserved material.

### 2.6 Hearing frequency and range

The length of the endosseous cochlear duct (lagena of some authors) is often used as a proxy for hearing range and sensitivity (Hanson et al., 2021; Walsh et al., 2009). We employ the methods used by Walsh et al., 2009 in estimating hearing parameters. Two metrics are required: endosseous cochlear duct (ECD; pars cochlearis) length and basicranial length (BCL). The dorsal limit of the former is usually defined by a marked constriction where the ECD meets the saccule (Walsh et al., 2009): preservation does not allow us to determinate this with absolute confidence in *Ceratosuchops* but we were still able to provide an estimate. The measurement tools in Meshmixer were employed for both spinosaurids.

The constraints of the BCL were not outlined in Walsh et al. (2009) but this measure is defined as the anteroposterior distance between the anterior limit of the basisphenoid (excluding the cultriform process of the parasphenoid) and the posterior margin of the occipital condyle (Dudgeon et al., 2020). In *Ceratosuchops*, the basisphenoid and parasphenoid are indiscernibly fused together; the anterior limit of the basisphenoid was herein defined by following an obliquely trending line from the anterior margin of the basipterygoid process pedicel towards the region of the pituitary fossa in lateral view.

The ECD was scaled to the BCL and log transformed. For hearing range, we followed the equation:

y=6104.3x+6975,

with *x* being the scaled and log‐transformed ECD, and *y* being the best hearing range in Hz. For mean hearing frequency we followed the equation:

y=3311.3x+4000.8,

with *x* being the scaled and log‐transformed ECD length, and *y* being the mean best hearing frequency in Hz.

We also calculated the hearing capabilities of several other theropods for further comparisons, using the STL models of theropod inner ears from Choiniere et al. (2021). These included: *Viavenator exonii* (MAU Pv LI 530), *Sinraptor dongi* (IVPP 10600), *Murusraptor barrosaensis* (MCF PVPH 411), and *Erlikosaurus andrewsi* (IGM 100.111). These are based on models from Lautenschlager et al. (2012), Paulina‐Carabajal and Currie (2012, 2017) and Paulina‐Carabajal and Filippi (2018). Approximate ECD dimensions were measured using the tools in Meshmixer, and BCL was estimated from published images (Lautenschlager et al. (2012): Figure 2a; Paulina‐Carabajal and Currie (2012): Figure 5a; Paulina‐Carabajal and Currie (2017): Figure 7.3; Paulina‐Carabajal and Filippi (2018): Figure 1d) using FIJI.

### 2.7 Olfactory acuity

The form and external limits of the olfactory bulbs are difficult to determine in the *Ceratosuchops* endocast, being located at the anterior end of the expanding olfactory tract and separated by a midline sulcus in dorsal view (see below). An olfactory ratio was calculated by measuring the longest diameters of the bulbs and cerebral hemisphere (regardless of orientation); this is expressed as a percentage following previous studies (Zelenitsky et al., 2009, 2011). Raw olfactory ratios should not be directly compared across taxa due to the influence of body size (Zelenitsky et al., 2009). As the mass of *Ceratosuchops* cannot be presently estimated but likely approximates that of *Baryonyx* (see above), we used the *Baryonyx* mass estimate employed in our REQ calculations and log‐transformed both metrics. These were subsequently plotted onto the graph produced by Zelenitsky et al. (2009: Figure 2). Given the use of both specimens in this analysis, we provisionally consider the latter result representative of the generalised baryonychine condition.

## 3 RESULTS

### 3.1 Baryonychine endocranial morphology

#### 3.1.1 Preservation

##### Baryonyx (NHMUK PV R9951)

The cerebral endocast is preserved in three disjunct pieces (Figure 2). A transverse break posterior to the dural peak and anterior to the trigeminal nerve (CN V) trunk separates the two main anterior and posterior portions, with a third located on the disarticulated left otoccipital. The left side of the anterior cerebral endocast is preserved as a natural cast and is visible macroscopically following the loss of the left frontoparietal and laterosphenoid. Unfortunately, the anterior four cranial nerve trunks, the left vestibulocochlear (CN VIII) and glossopharyngeal (CN IX) nerve trunks, and olfactory bulbs or tracts are not preserved.

Nevertheless, what remains of the endocast is well preserved, though a degree of plastic deformation affects the anterior braincase piece and the internal left otoccipital (as evidenced by the dorsoventrally compressed semicircular canals). The endosseous labyrinth of the right inner ear is well preserved and can be reconstructed in full (Figures 2, 4 and 5a–d), unlike its damaged and disjoined left counterpart, and this right‐side structure forms the basis of the structure's description and comparison.

##### Ceratosuchops inferodios (IWCMS 2014.95.1–3)

**FIGURE 3:**
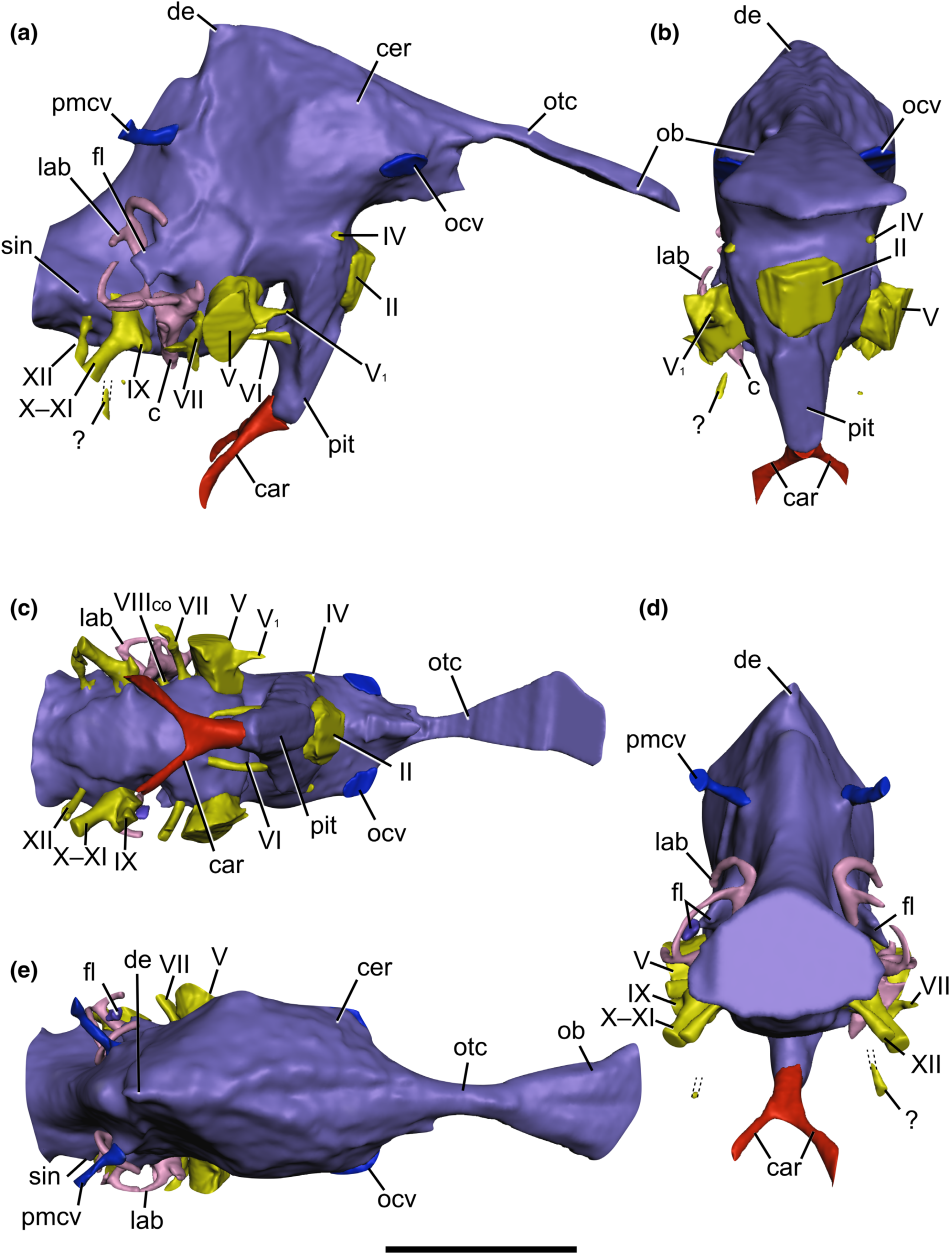
Cranial endocast of *Ceratosuchops inferodios* (IWCMS 2014.95.1–3), reconstructed from CT scans, in (a) right lateral, (b) anterior, (c) ventral, (d) posterior and (e) dorsal views. Vascular structures and endosseous labyrinth also depicted. Abbreviations: c, cochlea; car, cerebral internal carotid artery canal; cer, cerebral hemisphere; de, dorsal expansion; fl, floccular lobe; lab, endosseous labyrinth; ocv, orbitocerebral vein; pit, pituitary; pmcv, posterior middle cerebral vein canal; sin, blind dural venous sinus of the hindbrain; II, optic nerve canal; IV, trochlear nerve canal; V, trigeminal nerve canal; V_1_, ophthalmic nerve canal; VI, abducens nerve canal; VII, facial nerve canal, VIII_co_, cochlear ramus of the vestibulocochlear nerve; IX, glossopharyngeal nerve canal; X–XI, shared canal for the vagus, and accessory nerves, and accompanying vessels; XII, hypoglossal nerve canal; ?, potential accessory hypoglossal nerve or venous canal. Scale bar: 50 mm.

The endocast, once reassembled, is largely complete and includes all major cranial nerves and major vascular features (Figure 3). However, our effort to rearticulate the braincase has introduced three morphological artefacts to the endocast. These are as follows: (1) a divot on the posterior dorsal expansion, which was presumably smoother in life given the morphology of other theropods (Sampson & Witmer, 2007; Witmer & Ridgely, 2009); (2) the bilateral, transverse sinus‐like ridges posterior to the cerebral hemispheres do not represent cerebral topography but the supraoccipital‐parietal contacts; and (3) an artificial “step” between the ventral laterosphenoid‐prootic contacts which, when combined with the loss of a portion of the right dorsum sellae (exposing some of the right abducens (CN VI) nerve trunk in cross‐section), produces an anteriorly projecting ventral “lobe” on earlier iterations of the endocast near the right trigeminal (CN V) trunk. To avoid confusion or interpretation of this artefact as a genuine endocranial feature, this region of the endocast was bevelled in 3D view using the *Polygon* tool in ORS Dragonfly. This loss of bone and suboptimal laterosphenoid‐prootic contact also rendered it difficult to visualise the ventral border of the trigeminal nerve trunk, which was inferred based on the preserved margins of the trigeminal foramen.

Elsewhere, the floccular lobes could not be reconstructed in full: the left lobe exists as two disjointed pieces and the lateral extent of both cannot be determined. Image contrast issues mean that the posterior dorsal head veins were difficult to trace, while the secondary, more ventral passages for the hypoglossal (CN XII) nerve trunks could only be traced over a short distance. The left vestibulocochlear nerve trunks (CN VIII) could not be visualised.

**FIGURE 4:**
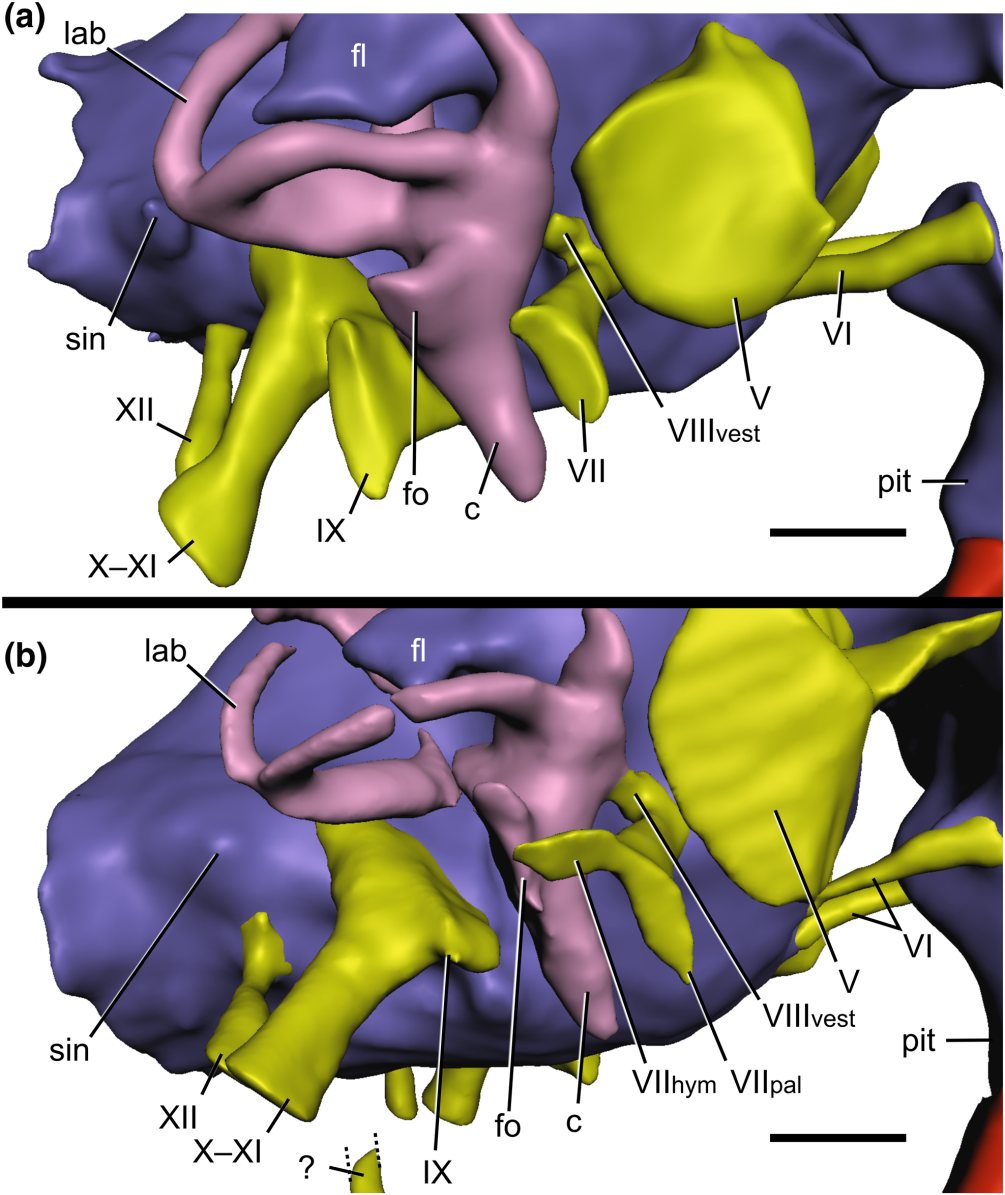
The baryonychine rhombencephalon, in right lateral views. (a) *Baryonyx walkeri*, (b) *Ceratosuchops inferodios*. Abbreviations: c, cochlea; fl, floccular lobe; fo, fenestra ovalis; lab, endosseous labyrinth; pit, pituitary; sin, blind dural venous sinus of the hindbrain; V, trigeminal nerve canal; VI, abducens nerve canal; VII_hym_, hyomandibular ramus of the facial nerve; VII_pal_, palatine ramus of the facial nerve; VIII_vest_, vestibular ramus of the vestibulocochlear nerve; IX, glossopharyngeal nerve canal; X–XI, shared canal for the vagus and accessory nerves, and accompanying vessels; XII, hypoglossal nerve canal; ?, potential accessory hypoglossal nerve or venous canal. Scale bar: 10 mm.

**FIGURE 5:**
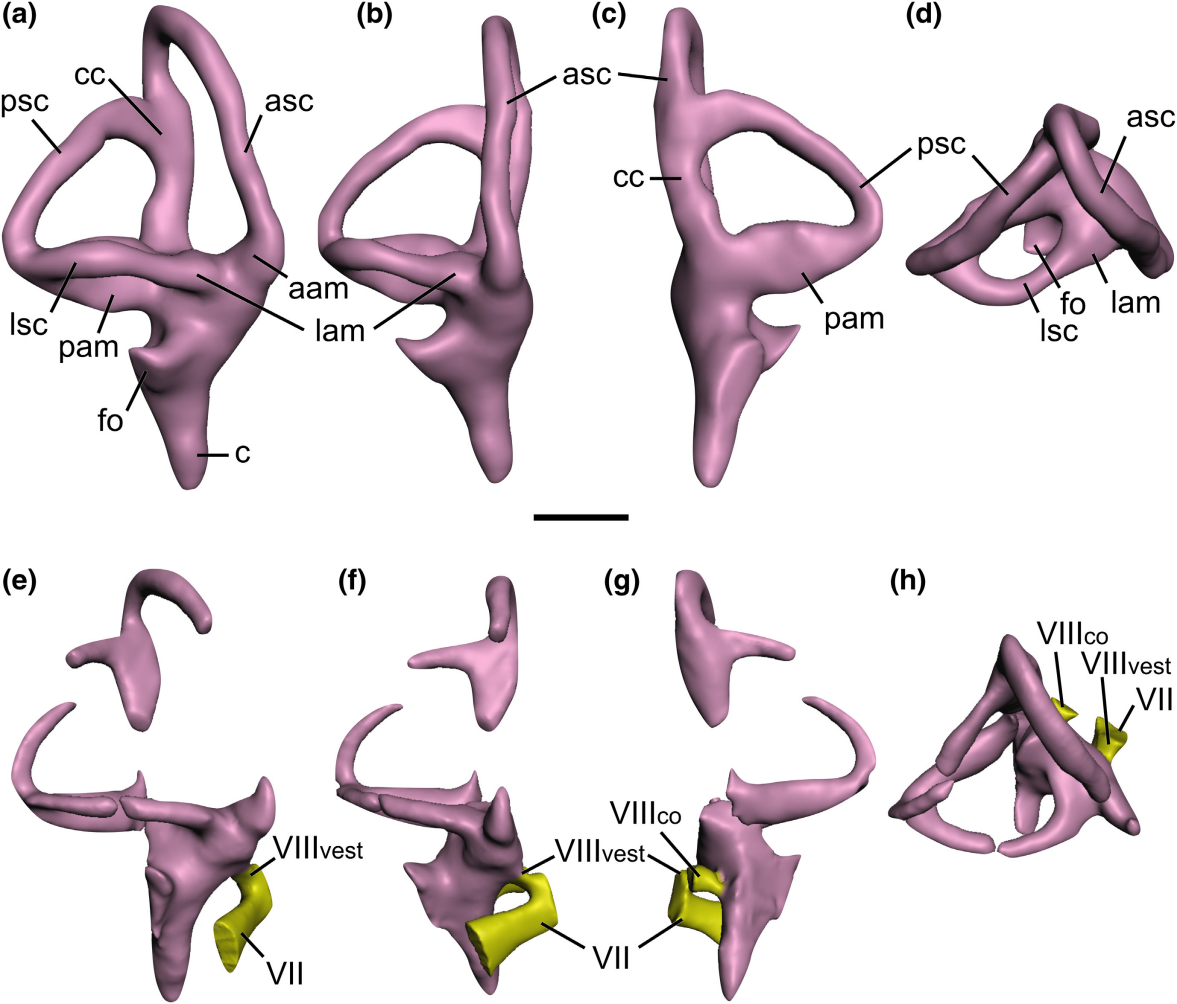
Baryonychine endosseous labyrinths. *Baryonyx walkeri* (a–d) and *Ceratosuchops inferodios* (e–h), in (a, e) lateral, (b, f) anterior, (c, g) posterior, (d, h) dorsal views. Note that the facial nerve (CN VII) trunk has been abbreviated in the *Ceratosuchops* model. Abbreviations: aam, anterior ampulla; asc, anterior semicircular canal; c, cochlea; cc, common crus; fo, fenestra ovalis; lam, lateral ampulla; lsc, lateral semicircular canal; pam, posterior ampula; psc, posterior semicircular canal; VII, facial nerve canal; VIII_co_, cochlear ramus of the vestibulocochlear nerve; VIII_vest_, vestibular ramus of the vestibulocochlear nerve. Scale bar 10 mm.

The endosseous labyrinths are partially preserved (Figures 3, 4 and 5e–h). The left side is missing its vestibule, cochlear duct and portions of the anterior and lateral semicircular canals, which could not be visualised due to the lack of contrast and presence of a substantial quantity of radiopaque matrix in the region. The right labyrinth is more complete and forms the basis of the structure's description and comparison, although the midsection of the anterior semicircular canal (within the prootic) could not be segmented (this was also due to the presence of radiopaque matrix obscuring its path). The posterior margin of the cochlear duct could not be reconstructed due to disarticulation of the prootic and otoccipital.

#### 3.1.2 Endocranial morphology

Baryonychine endocasts (Figures 2 and 3) conform to the general morphology of other non‐coelurosaurian theropods, the details of which are discussed below, and corroborate previous suggestions of endocranial conservatism amongst the taxa concerned (Hopson, 1979; Sampson & Witmer, 2007). As is typical for most non‐maniraptoriform sauropsids, the brain did not fill the endocranial cavity, with much obscured by a dural envelope and its extensive venous sinuses (Hopson, 1979; Sampson & Witmer, 2007; Sedlmayr, 2002; Witmer et al., 2008; Witmer & Ridgely, 2009). To facilitate interspecific comparisons, the endocasts will be oriented with the lateral semicircular canal assuming a horizontal path.

The olfactory tract is well preserved in *Ceratosuchops*. It is an elongate, narrow structure that expands anteriorly towards the olfactory bulbs. The anterior portion is indented along its dorsal midline by a shallow sulcus, as in *Irritator* (Schade et al., 2020), likely marking the position of the olfactory bulbs. The tracts are in line with the forebrain in both these specimens, and their elongate proportions conform to the plesiomorphic archosaurian condition present in many non‐avian theropods (Bever et al., 2013; Franzosa, 2004); though incomplete, the base of the olfactory tract is also present in *Baryonyx*, and a similar morphology is thus inferred. The spinosaurids lack evidence of medial separation of their respective bony olfactory tracts, as in *Majungasaurus* (Sampson and Witmer, 2007) and various allosauroids excluding *Acrocanthosaurus* (Eddy & Clarke, 2011; Franzosa & Rowe, 2005; Paulina‐Carabajal & Currie, 2012).

Although the brain does not completely occupy the endocranial cavity, the portion of the endocast corresponding to the telencephalon is considered to approximate the underlying brain contours, especially laterally (Sampson & Witmer, 2007; Sedlmayr, 2002; Witmer et al., 2008); the cerebral hemispheres can be clearly observed. The studied baryonychines possess the relatively small and unexpanded ancestral morphology typical of non‐maniraptoriform theropods (Franzosa, 2004; Larsson et al., 2000; Rauhut, 2003). Despite the limited expansion, the widest part of the endocast appears located at the level of the cerebral hemispheres, as is typical for fossil reptiles (Hopson, 1979) and observed in such theropods as *Irritator* (Schade et al., 2020), abelisaurids (Paulina‐Carabajal & Filippi, 2018; Sampson & Witmer, 2007), *Murusraptor* (Paulina‐Carabajal & Currie, 2017) and allosauroids such as *Allosaurus* (Rogers, 1998) and *Sinraptor* (Paulina‐Carabajal & Currie, 2012).

The forebrain and hindbrain are generally horizontally oriented in both baryonychines, as is typical for non‐coelurosaurian theropods and also early‐branching coelurosaurs such as tyrannosaurids (Witmer & Ridgely, 2009). A nuance is that the forebrain is directly slightly anteroventrally along its length in *Ceratosuchops*. Differences are noted in the degree of flexure between the baryonychine endocasts. The estimated cephalic and pontine flexures sensu Hopson (1979) are presented in Table 1 and suggest that the midbrain region is more strongly angled in *Ceratosuchops*, although precise measurement of these angles is problematic due to the obscuring nature of the surrounding dural envelope (Witmer & Ridgely, 2009). Midbrain angulation of around 45–60° is observed in non‐coelurosaurian theropods generally (Hopson, 1979; Witmer & Ridgely, 2009).

In contrast to *Baryonyx*, the dorsal surface of the *Ceratosuchops* endocast preserves a narrow midline sulcus (Figure 3e). This likely does not represent the interhemispheric fissure, a feature typically obscured by the dorsal longitudinal sinus in non‐avian theropods (Cerroni & Paulina‐Carabajal, 2019; Hopson, 1979; Witmer et al., 2008) but reported in various maniraptorans (Balanoff et al., 2009, 2014; Hattori et al., 2021; Lautenschlager et al., 2012). Instead, these potentially represent anteriorly diverging venous channels emanating from the dorsal expansion.

The dorsal expansion of the endocast – an eminence just posterior to the cerebrum – has been thought to house (at least in part) the pineal apparatus in non‐coelurosaurian theropods, although the expansion is absent in more basal theropods (Sampson & Witmer, 2007; Witmer & Ridgely, 2009). However, recent work on extinct and extant turtles suggests that a cartilaginous origin, rather than one relating to the pineal gland also merits consideration (Werneburg et al., 2021), as does a venous interpretation as the dorsal expansion is associated with a variety of clearly venous structures in a variety of extinct and extant diapsids (Porter & Witmer, 2015, 2020; Witmer et al., 2008; Witmer & Ridgely, 2009). The dorsal expansion is pronounced and ascends above the dorsal margin of the forebrain in *Baryonyx*, in contrast to *Ceratosuchops*. As such, the dorsal margin of the latter's forebrain assumes a largely linear trend in lateral view, contrasting against the more concave margin in *Baryonyx*. As in *Majungasaurus* and *Allosaurus* (Sampson & Witmer, 2007), the dorsal expansion in *Baryonyx* is located within the parietals, whereas *Ceratosuchops* recalls the condition in tyrannosaurids, where the apex is situated at the parietal‐supraoccipital suture (Bever et al., 2013). Variation in the development of the dorsal expansion has been noted in *Tyrannosaurus*; however, it is likely that this structure is non‐homologous with that of non‐coelurosaurian taxa (Witmer & Ridgely, 2009). Damage to this region in *Irritator* (Schade et al., 2020) precludes accurate comparisons with the baryonychines.

The dorsal expansion's peak is approximately level with the trigeminal nerve trunk in both baryonychines, recalling the condition in *Majungasaurus* (Sampson and Witmer, 2007), *Murusraptor* and *Tyrannosaurus* (Paulina‐Carabajal & Currie, 2017), whereas it is more anteriorly positioned in various allosauroids and ceratosaurs (Paulina‐Carabajal & Currie, 2012, 2017; Rogers, 1998). Conversely, the peak is more posteriorly positioned in *Alioramus* and *Viavenator*, being respectively level with the facial (CN VII) nerve trunk and floccular lobe (Bever et al., 2013; Paulina‐Carabajal & Filippi, 2018). The dorsal expansion has also been used to approximate the size of the midbrain region in theropods, with many non‐coelurosaurian tetanurans and ceratosaurs possessing elongated midbrain regions (Paulina‐Carabajal & Currie, 2017). The sampled baryonychines follow this trend (Table 2), although the elongation is less marked compared to such forms as *Majungasaurus* or *Allosaurus*.

**TABLE 2:** Approximate elongation of the midbrain region in select theropods. Midbrain and medulla lengths follow Paulina‐Carabajal and Currie (2017): midbrain length measured from the anterior border of the floccular lobe to the peak of the dorsal expansion; medulla length measured from the foramen magnum to the trigeminal nerve (CN V) trunk.

	Midbrain length (mm)	“Medulla” length (mm)	Midbrain: “medulla” ratio	References
*Sinosaurus*	50.2	52.5	0.96	Xing et al. (2014: Fig. 6A)
*Majungasaurus*	52.8	36.2	1.46	Sampson and Witmer (2007: Figure 18A)
*Viavenator*	49.8	41.7	1.19	Paulina‐Carabajal and Filippi (2018: Figure 4a)
*Baryonyx*	61.6	46.9	1.31	This paper
*Ceratosuchops*	61.1	49.9	1.22	This paper
*Allosaurus*	58.9	38.6	1.53	Witmer and Ridgely (2009: Figure 4b)
*Acrocanthosaurus*	55.6	44	1.26	Franzosa and Rowe (2005: 2B)
*Murusraptor*	33.5	32	1.05	Paulina‐Carabajal & Currie (2017: Figure 7.4)
*Tyrannosaurus*	40.7	50.9	0.8	Witmer and Ridgely (2009: Figure 4c)
*Erlikosaurus*	20.4	27.7	0.74	Lautenschlager et al. (2012: Figure 3a)
*Deinonychus*	16.3	20.7	0.79	Witmer and Ridgely (2009: Figure 4e)

*Note*: We note that the medulla was likely obscured by the dural envelope (see main text), and the medulla measurement of Paulina‐Carabajal and Currie (2017) instead approximates the length of the rhombencephalon. Measurements for non‐spinosaurids taken from images using FIJI.

The optic lobes (optic tecta) in the baryonychines are imperceptible, recalling the plesiomorphic sauropsid condition (Franzosa, 2004; Schade et al., 2020); this is also the case in *Irritator* (Schade et al., 2020). These lobes are equivocally visible in some *Tyrannosaurus* specimens, and a trend whereby these structures become increasingly obvious is observed within coelurosaurs on the line to birds (Witmer & Ridgely, 2009). The course of the transverse sinus and middle cerebral vein can be used to identify the gross position of the optic lobes and cerebellum, given that these structures pass between these brain regions in extant sauropsids (Sampson & Witmer, 2007; Witmer & Ridgely, 2009). The transverse sinus is difficult to differentiate in the *Baryonyx* endocast, while an artificial ridge in *Ceratosuchops* corresponds to the rearticulated laterosphenoid‐prootic and supraoccipital‐parietal contacts. There is also no clear evidence of a canal or foramen corresponding to the passage of the middle cerebral vein in either baryonychines (the implications of which are discussed below). In sum, the positions of the optic lobes remain obscured.

The cast of the baryonychine pituitary (hypophyseal) fossa, which mostly housed the pituitary gland (Sampson & Witmer, 2007) but likely contained various other soft tissue structures (Hopson, 1979), is a largely vertically oriented structure located just anterior to the level of the dural peak when viewed laterally. It produces a pair of dorsal and ventral posterior projections in the baryonychines, imparting a “wavy” posterior margin in lateral view. In *Irritator*, the posterior margin is more uniformly convex (Schade et al., 2020), although variation in the shape of the pituitary fossa is known amongst well sampled theropods such as *Tyrannosaurus* (Bever et al., 2013). A minor degree of posterior angulation is also noted in the *Irritator* pituitary, which would follow the conservative theropod pattern (Bever et al., 2013); baryonychines, however, do not appear to deviate substantially from the vertical.

Within the region of the pituitary fossa, *Ceratosuchops* possesses the plesiomorphic cavernous sinus present in archosaurs and many non‐coelurosaurian theropods through which pass the trochlear (CN IV) and abducens (CN VI) nerves, their associated vasculature, and likely the encephalic branches of the cerebral carotid artery (Bever et al., 2011; Sampson & Witmer, 2007; Witmer & Ridgely, 2009). This feature could not be discerned in *Baryonyx* due to preservation.

Discrimination of the posterior regions of the brain, including the cerebellum, medulla and pons, is difficult in the baryonychine endocasts, and it most probably resembled other basal theropods and archosaurian outgroups in possessing extensive dural venous sinuses that obscured morphological details (Sampson & Witmer, 2007; Sedlmayr, 2002). We note, however, that the ventral morphology of the rhombencephalon (i.e. the region posterior to the trigeminal (CN V) nerve trunk in lateral view) is markedly more convex in *Baryonyx* (Figure 2a) than in *Ceratosuchops* (Figure 3a) when viewed laterally, and recalls the condition observed in *Irritator*.

The floccular lobes (part of the cerebellum) are distinguishable, however, as is typical for theropods (Franzosa, 2004) and dinosaurs more generally (compared to extant non‐avian reptiles) (Witmer et al., 2003). The lobes themselves, best visualised on the well‐preserved right side of the *Baryonyx* endocast, display the mediolaterally thin and somewhat triangular/tabular lateral morphology of many non‐maniraptoriform theropods, lacking the bulbous derived condition (Franzosa, 2004; Paulina‐Carabajal & Currie, 2017; Witmer & Ridgely, 2009). Like *Irritator*, these project posterolaterally from the endocast, passing the plane of the anterior semicircular canal to occupy the space delineated by these vestibular structures, as in many theropods other than adult tyrannosaurids (Witmer & Ridgely, 2009), whose floccular lobes barely project into this space (but may not truly represent the size of the neural structure in life; see Witmer & Ridgely, 2009).

The lateral extent of the lobes could not be reliably reconstructed in *Ceratosuchops*; however, the floccular lobes of *Baryonyx* terminate medially to the lateral semicircular canal when viewed dorsally (Figure 2e). In contrast, said lobes were described as notably large in *Irritator*, additionally exhibiting lateral extent to reach the level of the lateral semicircular canal (Schade et al., 2020). Floccular lobes that reach the level of the lateral semicircular canal are observed in such theropods as *Velociraptor* (which also displays the more bulbous morphology typical of coelurosaurs) (King et al., 2020).

Two small, conical, lateral protrusions are present bilaterally on the posterior endocasts of both baryonychines, posterior to the endosseous labyrinth and dorsal to the hypoglossal (CN XII) canals. Equivalently positioned “blind dural sinus of the hindbrain” have been documented in an indeterminate theropod (Knoll et al., 1999), *Tyrannosaurus* (Witmer & Ridgely, 2009) and the sauropod *Spinophorosaurus* (Knoll et al., 2012), and a similar feature was tentatively identified as an “endolymphatic duct” in *Murusraptor* (Paulina‐Carabajal & Currie, 2017). Rounded protuberances in this region are nevertheless noted in many archosaurs, where they have been interpreted as diverticula of the longitudinal sinus (Hopson, 1979).

#### 3.1.3 Cranial nerve trunks

Cranial nerve trunk organisation is highly conserved within Dinosauria (Hopson, 1979; Witmer & Ridgely, 2009), and the baryonychine endocasts do not deviate from this general pattern (Figures 1, 2, 3). As above, however, cranial nerves I–IV are unfortunately not preserved in *Baryonyx*, although the more posterior nerve trunks can all be readily distinguished. We reiterate previous works in noting that veins, as well as nerves, likely passed through the cranial nerve foramina, as is the case in extant archosaurs (Sampson & Witmer, 2007; Sedlmayr, 2002), such that segmented canals likely contained both soft tissue structures.

In *Ceratosuchops*, both the olfactory bulbs and tract and optic nerve (CN II) trunk are situated medially and project anteriorly from the endocast. The latter is large and undivided in contrast to the condition present in some abelisaurids, whose optic nerve trunk may be subdivided by the calcified interorbital septum (Sampson & Witmer, 2007). The small, ovate trochlear nerve (CN IV) trunks are located ventral to the cerebral hemispheres. These are usually situated dorsally to the oculomotor nerve (CN III) trunks in archosaurs (Hopson, 1979); however, independent foramina for the latter could not be distinguished. The trochlear nerve trunks identified in *Irritator* (Schade et al., 2020; Sues et al., 2002) are too dorsally located to correspond to this cranial nerve, which always passes behind and then ventral to the optic lobe in extant taxa (Witmer & Ridgely, 2009). Instead, these most likely correspond to passages for orbitocerebral veins, as observed in *Majungasaurus* (Sampson & Witmer, 2007).

The trigeminal nerve (CN V) trunks possess a large diameter and project laterally from the ventrolateral endocast surface in both baryonychines. This structure is undivided and exits the lateral braincase via a single foramen in both specimens. This mirrors the condition in *Irritator* (Sues et al., 2002) and most carcharodontosaurids (Brusatte et al., 2010), indicating an extracranial position of the trigeminal (Gasserian) ganglion (Witmer et al., 2008). In contrast, most tetanurans possess the derived condition whereby separate foramina are present for the transmission of the trigeminal's ophthalmic (CN V_1_) and maxillomandibular (CN V_2, 3_) rami (this demonstrating an intracranial position of the ganglion) (Franzosa, 2004; Witmer et al., 2008; Witmer & Ridgely, 2009). The partial canal for the ophthalmic ramus could be segmented in *Ceratosuchops*: it splits from the lateral (extracranial) part of the trigeminal nerve trunk and extends anteriorly within a shallow sulcus on the ventrolateral surface of the laterosphenoid.

The horizontal abducens nerve (CN VI) canals are located ventrally and medially to the trigeminal nerve (CN V) trunks and are present anterior to the latter to open into the dorsal pituitary fossa. This positioning within the pituitary fossa in the baryonychines is plesiomorphic for theropods; it has been reported in *Irritator* and various allosauroids (Franzosa, 2004) and differs from the derived and more laterally projecting coelurosaur condition (Bever et al., 2011; Witmer & Ridgely, 2009). This more lateral course results from the loss of the above‐mentioned cavernous sinus in members of the latter clade, although it is present in some tyrannosauroids (Bever et al., 2011, Witmer & Ridgely, 2009).

The facial nerve (CN VII) trunk becomes dorsoventrally expanded as it approaches its lateral exit through the prootic. Dorsoventral elongation of the associated external foramen has been recovered as a spinosaurid synapomorphy in some studies (Barker et al., 2021). The associated palatine and hyomandibular rami most probably diverged upon exiting the external facial nerve foramen, and thus indicate an extracranial position of the associated geniculate ganglion that is typical of theropods bar *Alioramus* (Bever et al., 2011). Indeed, sulci on the lateral surface of the prootic, whilst difficult to discern in *Baryonyx*, are visible in the better‐preserved *Ceratosuchops* braincase and bear testament to this extracranial condition.

Dorsal to the facial nerve trunk is the vestibular ramus of the vestibulocochlear nerve (CN VIII_vest_), which shares a common internal foramen with the former in *Ceratosuchops* (and probably *Baryonyx* as well). This ramus courses dorsolaterally towards the vestibule of the ipsilateral endosseous labyrinth in both baryonychines (Figure 4). The diminutive cochlear ramus of the vestibulocochlear nerve (CN VIII_co_) trunk is located posterior to the facial nerve trunk, projecting laterally towards the endosseous labyrinth. This ramus appears to split laterally as it approaches the labyrinth in *Ceratosuchops* (Figure 5g).

The dorsoventrally elongate glossopharyngeal nerve (CN IX) trunk and more tubular vagal canal for the vagus (CN X) and accessory nerve (CN XI) trunks diverge from a common protruding feature on the hindbrain of the endocast: the metotic foramen. The foremost nerve trunk transmits laterally posterior to the fenestra ovalis and is level with the cochlear duct of the endosseous labyrinth. The vagus and accessory nerve trunks, as well as a probable small jugular vein (Bever et al., 2013; Sampson & Witmer, 2007), take a more posterior course and open on the occiput. This organisation, and notably the posterior diversion of the vagal canal, is indicative of the subdivision of the metotic foramen by the anteromedial aspect of the otoccipital (or, more specifically, its crista tuberalis) (Bever et al., 2013; Gower & Weber, 1998; Sampson & Witmer, 2007). *Baryonyx* and *Ceratosuchops* thus possess the derived theropod condition (Rauhut, 2003; Sampson & Witmer, 2007), which is also observed in *Irritator*. The subdivision of the cavum metoticum is incomplete in the baryonychines and the crista tuberalis does not meet the lateral endocast wall; the medial aperture of the cavum is thus undivided, as in most archosaurs (Bever et al., 2013).

Only a single hypoglossal nerve (CN XII) trunk is bilaterally present in *Baryonyx* (Figure 2d): this opens within a common fossa on the occiput alongside the vagal canal. Ceratosuchopsin spinosaurids, in contrast, possess a smaller third external foramen located ventrally to the vagal and hypoglossal foramina (and thus display three external foramina penetrating the occiput) (Barker et al., 2021). The path of the corresponding third canal can only be traced a short distance in *Ceratosuchops*, and its identity is unclear (Figure 3d); it may represent an accessory hypoglossal nerve trunk or a venous canal (Witmer & Ridgely, 2009). Three canals can also be seen in *Irritator* (M. Schade pers. comms, 2022), contra Sues et al. (2002) and Schade et al. (2020). Elsewhere among Theropoda, single hypoglossal canals/foramina have been noted in abelisaurids (Cerroni & Paulina‐Carabajal, 2019; Paulina‐Carabajal & Filippi, 2018; Sampson & Witmer, 2007) and *Alioramus* (Bever et al., 2013), whilst two are observed in *Dilophosaurus* (Marsh & Rowe, 2020) and *Allosaurus* (Hopson, 1979), though we note that Franzosa (2004) scores the latter taxon as possessing only a single foramen in specimen UMNH VP 18055 (previously UUVP 5961). Two hypoglossal canals are also present in various maniraptoriforms (Franzosa, 2004; Lautenschlager et al., 2012).

The presence of three external foramina on the occiput was deemed synapomorphic for megalosauroids in a previous phylogenetic analysis, the spinosaurid condition whereby two are present being regarded as a reversal (Carrano et al., 2012). The new information from *Irritator* and ceratosuchopin baryonychines (sensu Barker et al., 2021) would instead suggest that three passages is the more probable ancestral state amongst the spinosaurids, with the two canals in *Baryonyx* potentially representing an autapomorphic condition. However, we note here that polymorphism in hypoglossal foramen and internal canal number has been reported in *Tyrannosaurus* (Bever et al., 2013; Witmer & Ridgely, 2009), a well‐sampled theropod taxon, and the character may not be as phylogenetically relevant as previously assumed.

#### 3.1.4 Vascular structures

As mentioned above, a pair of the large, ovoid channels located on the anteroventral surface of the cerebral hemispheres corresponds to passages for the orbitocerebral veins. Posterodorsally, the paired canals of the posterior middle cerebral veins (=transversooccipital veins) adjoin the endocast posterior to the dural peak in both baryonychines, as is common for theropods. The canals are short and posterodorsally trending, opening on the occipital surface of the supraoccipital bone near its contact with the parietal.

Laterally, the lack of an obvious independent foramen in the laterosphenoid for the anterior middle cerebral vein (=transversotrigeminal vein) in either baryonychine suggests this vessel passed through the trigeminal foramen (or, potentially the prootic‐laterosphenoid suture), in contrast to the condition present in many other saurischians (Rauhut, 2003).

Ventrally, the canals of the internal carotid arteries unite anteriorly prior to entering the posteroventral pituitary fossa, imparting a V‐shaped morphology in the baryonychines when viewed ventrally. Such organisation is unusual among non‐coelurosaurian theropods: certain ceratosaurs, allosauroids and early neotheropods possess carotid canals that enter the pituitary fossa separately (Franzosa, 2004; Marsh & Rowe, 2020; Sampson & Witmer, 2007). The condition in *Irritator* cannot be ascertained (Schade et al., 2020), but that in *Baryonyx* and *Ceratosuchops* recalls the condition observed in *Tyrannosaurus* and several other coelurosaurs (Franzosa, 2004), as well as *Giganotosaurus* (Paulina‐Carabajal & Canale, 2010).

#### 3.1.5 Endosseous labyrinth

The inner ear can be grossly subdivided into two mechanosensory organs that are respectively responsible for the detection of head movements and sound – the vestibular labyrinth (composed of the three semicircular canals, the utriculus and sacculus) and cochlea (which includes the cochlear and perilymphatic ducts) (Bronzati et al., 2021; Hanson et al., 2021; Pfaff et al., 2019; Wever, 1978; Witmer et al., 2003). Despite representing a (slightly) smaller individual, the *Baryonyx* inner ear is a slightly larger structure compared to the reassembled organ of *Ceratosuchops*, but the disarticulation experienced by the latter's braincase may have impacted some of its dimensions (Figure 5). Nevertheless, the semicircular canals are also slightly larger in diameter in *Baryonyx*.

The baryonychine semicircular canals are generally thin structures organised roughly orthogonally relative to one another, as seen in other theropods (Sampson & Witmer, 2007; Witmer & Ridgely, 2009). The vertical semicircular canals are asymmetrical – a common feature among theropods including *Irritator* (Bever et al., 2013; Schade et al., 2020). The anterior semicircular canal is tall – a typical morphotype amongst avemetatarsalians (Hanson et al., 2021) – and like other dinosaurs (Sampson & Witmer, 2007), it is the longest of the three canals in both baryonychines. These possess the elliptical morphology observed in other noncoelurosaurian theropods, lacking the anterior and posterodorsal expansion noted in tyrannosaurids and other coelurosaurs (Witmer & Ridgely, 2009). It rises above the dorsal level of the posterior canal prior to deflecting ventrally to meet the common crus between the pair. The anterior canal remains in line with the common crus, such that latter does not show the twisting seen in various coelurosaurs (Sampson & Witmer, 2007; Witmer & Ridgely, 2009). It maintains a near‐uniform diameter throughout most of its length; however, a slight ventral dilation – the anterior ampulla – can be observed in both of the Wealden Supergroup spinosaurids where the canal contacts the vestibule.

The shorter posterior semicircular canal in both baryonychines forms a simple, near‐vertically oriented arc, generally comparable to the non‐coelurosaurian condition (Bever et al., 2013). Its parasagittal orientation, which helps create a sub‐triangular space between the three semicircular canals in lateral view, is representative of the plesiomorphic theropodan condition observed amongst various non‐maniraptoriform theropods (Franzosa, 2004), including *Irritator* (Schade et al., 2020). Baryonychines also lack the anterior bowing of the posterior canal observed in tyrannosaurids and most other coelurosaurs (Witmer & Ridgely, 2009).

The lateral semicircular canal in *Baryonyx*, like that of *Irritator*, is not particularly bowed laterally in dorsal view: its anterior portion assumes a largely linear initial trajectory. This is again typical of non‐coelurosaurian theropods (Bever et al., 2013), and the canal in both spinosaurids only “hooks” towards the secondary common crus in their posterior halves. *Allosaurus* produces a similar (if not slightly more exaggerated) posterior “hook” (Rogers, 1998; Witmer & Ridgely, 2009). In comparison, the lateral semicircular canal of *Ceratosuchops* is more uniformly bowed along its length, although the arc through which it sweeps is not as broad as that of various coelurosaurs (Witmer & Ridgely, 2009). Nevertheless, distinction between the posterior and lateral semicircular canals is impossible to determine in posterior view in any of the above‐mentioned spinosaurids, a characteristic of non‐maniraptoran theropods (Witmer & Ridgely, 2009).

The vestibule is typically archosaurian (some exceptions not withstanding) in failing to extend dorsally above the level of the lateral semicircular canal (Bever et al., 2013; Sampson & Witmer, 2007; Witmer & Ridgely, 2009). The ventrally projecting cochlea is relatively long, as in archosaurs generally (Hanson et al., 2021), and accounts for approximately two‐thirds of the dorsoventral height of the vestibular labyrinth in both baryonychines. Intriguingly, this is different from the subequal proportions described for *Irritator* (Schade et al., 2020) and the relatively short cochlea of abelisaurids (Cerroni & Paulina‐Carabajal, 2019). A subtle degree of medial inclination is also observed in *Baryonyx* and *Ceratosuchops* when viewed anteriorly, as in *Irritator* (Schade et al., 2020) and tyrannosaurids (Witmer & Ridgely, 2009). In comparison, the medial inclination of the cochlea is more exaggerated in *Sinraptor* and *Murusraptor* (Choiniere et al. (2021): Data S2). A small, dorsally situated lateral projection on the cochlear duct in both baryonychines marks the passage from the fenestra ovalis, through which the columella passes.

#### 3.1.6 Basicranial pneumaticity

Details of the braincase pneumaticity will be presented elsewhere; here, we include brief comments given their effects on hearing capabilities, which are discussed below. Relative to that of some other theropods such as tyrannosaurids (Witmer & Ridgely, 2009), pneumaticity is only subtly developed in the *Baryonyx* and *Ceratosuchops* braincase: pneumatic structures include indentations on the lateral basisphenoid for the lateral tympanic recess, and deeper subsellar and basisphenoidal recesses within the cultriform process and basisphenoid, respectively.

An indentation on the posterior basioccipital and ventral to the occipital condyle is present in both taxa, which varies substantially in form and development. This was incorrectly referred to as a “subcondylar recess” in *Ceratosuchops* in Barker et al. (2021); however, such a recess usually refers to paired structures and is only present in a few theropod clades such as tyrannosaurids and ornithomimosaurs (Witmer, 1997). It remains unclear whether these indentations pertain to an excavation by a pneumatic system or are simply epiphenomena related to the elevation of the muscular ridges that delimit them laterally.

## 4 DISCUSSION

The cranial endocasts of the Wealden Supergroup baryonychines provide insight into the early evolution and anatomy of the spinosaurid endocranium. Like *Irritator* (Schade et al., 2020), both *Baryonyx* and *Ceratosuchops* possess neuroanatomical features typical of their phylogenetic position as non‐coelurosaurian tetanurans. These include the possession of weakly demarcated brain regions, prominent cranial flexures, mediolaterally thin, “tabular” floccular lobes, and asymmetrical vertical semicircular canals.

With the above‐described caveats regarding the body mass estimation used in our assessment of relative encephalisation, the calculated REQ for *Baryonyx* is approximately between 1.2 (REQ_37%_) and 1.6 (REQ_50%_). Some question exists on the utility of encephalisation quotients (Balanoff & Bever, 2020); nevertheless, we note that the degree of encephalisation calculated for large predatory theropods is generally comparable, the exception being select tyrannosaurids (Table 3). This, combined with the conservative nature of non‐coelurosaurian theropod endocranial morphology, implies comparable cognitive capacity and behavioural sophistication among the taxa concerned.

**TABLE 3:** Reptile Encephalisation Quotients (REQ) calculated for a range of theropods.

Taxon	EV (cm^3^)	EV (cm^3^) 37%	EV (cm^3^) 50%	Mbd (g)	REQ 37%	REQ 50%
*Baryonyx*	150.5	55.7	75.3	2,011,000	1.2	1.6
*Carnotaurus*	169.8	62.8	84.9	1,419,000– 1,743,000	1.4–1.6	1.9–2.2
*Majungasaurus*	106.4	39.4	53.2	1,130,000	1.14	1.54
*Ceratosaurus*	–	–	–	–	1.2	1.7
*Allosaurus*	169–188	62.5–69.5	84.5–93.9	1,400,000–2,300,000	1.3–1.8	1.8–2.4
*Sinraptor*	95	35.1	47.5	1,700,000	0.8	1.1
*Giganotosaurus*	275	101.7	137.5	7,000,000	1.1	1.4
*Murusraptor*	148.2	54.8	74.1	1,551,000	1.33	1.8
*Tyrannosaurus*	414.2	153.2	207.1	5,654,000–7,000,000	1.8–1.6	2.5–2.2
*Gorgosaurus*	128.9	47.7	64.5	1,100,000	1.4	1.9

*Note*: Modified from (Cerroni & Paulina‐Carabajal, 2019). Note that the endocranial volumes (EV) at 37% and 50% of *Giganotosaurus* were misreported in Cerroni and Paulina‐Carabajal (2019). Mbd, body mass.

Some differences in endocranial and neurovascular morphology are present between *Ceratosuchops* and *Baryonyx*. These include the number of hypoglossal nerve (CN XII) canals (and their associated external foramina), the cranial flexure angles, the relative position of the dorsal expansion, the shape of the dorsal margin in the regions of the telencephalon and ventral margin of the rhombencephalon, the shape of the lateral semicircular canal, and the dimensions of the inner ear. It is not clear whether any of these lend support to the osteological characters used to differentiate the Wealden Supergroup baryonychines by Barker et al. (2021): endocasts of better‐sampled theropods such as *Allosaurus* (Hopson, 1979) and *Tyrannosaurus* (Witmer & Ridgely, 2009) have been described as “very similar” or “[showing] little variation”. Variation, when present, is possibly taphonomic or ontogenetic in nature, but studies examining this variation is rare (Lautenschlager & Hübner, 2013) or speculative (McKeown et al., 2020), and our understanding of its influence between and within taxa remains preliminary (Hu et al., 2021).

### 4.1 Inferred sensory capabilities of the Wealden Supergroup spinosaurids

#### 4.1.1 Vision and gaze stabilisation

As with many other studied theropods (Witmer & Ridgely, 2009), the imperceptible optic lobes in both baryonychine endocasts provide little information regarding visual acuity or sensitivity. The floccular lobes, however, may provide some information on the visual system. Integrating information from the latter as well as the vestibular systems of the inner ear (Voogd & Wylie, 2004), the floccular lobes regulate gaze stabilisation via compensatory movements of the eyes in response to rotation of the head (vestibulo‐ocular reflex, VOR), help track moving objects within the field of view (smooth pursuit), and (in some taxa) stabilise the head via recruitment of the cervical musculature (vestibulo‐collic reflex, VCR) (Ferreira‐Cardoso et al., 2017; Ito, 1982; Witmer et al., 2003).

Relative floccular lobe size has been used (in part) to reconstruct theropod ethology, where enlargement has been qualitatively correlated with increased capacity for gaze stabilisation (Cerroni & Paulina‐Carabajal, 2019; King et al., 2020; Lautenschlager et al., 2012). The relatively large floccular lobe in *Irritator* (compared to other non‐coelurosaurian theropods but not many coelurosaurs) was regarded as evidence of improved performance of the VOR and VCR systems (Schade et al., 2020). However, the size of this structure appears to correlate negatively with body size in theropods (Paulina‐Carabajal et al., 2021). Size may also reflect differing ontogenetic status, as has been noted during tyrannosaurid ontogeny, for example, and the small condition in mature individuals may not necessarily reflect of the size of the neural structure in life (Witmer & Ridgely, 2009). Indeed, rather than being indicative of differing ecologies, the influence of allometry or ontogeny could explain the difference in floccular lobe size in *Baryonyx* and *Irritator*, whose estimated skull lengths (*Irritator*, 600 mm; *Baryonyx*, 910 mm; Sues et al., 2002; Therrien & Henderson, 2007) tentatively suggest differing ontogenetic status. We note, however, that the assessment of ontogenetic status in non‐coelurosaurian theropods is complicated (Griffin et al., 2020), and as a result, the relative age of the above spinosaurids has not been rigorously determined. Further clouding the interpretation of floccular lobe size is the fact that the reconstructed structures may not be indicative of the neural tissues themselves, since additional tissues were likely also present alongside the floccular lobes (Ferreira‐Cardoso et al., 2017; Walsh et al., 2013).

The more complete right floccular lobe of *Baryonyx* is grossly comparable to other non‐coelurosaurian tetanurans in shape and extent. With the above caveats in mind and assuming the preserved lobe contained comparable amounts of neural tissue per unit volume, this similarity crudely implies *Baryonyx* possessed similarly developed gaze stabilisation mechanisms, and that these were potentially less developed than in *Irritator*.

Although beyond the scope of this study, some preliminary inferences regarding the visual capabilities of *Ceratosuchops* can be additionally made via the skeletal elements. Rearticulation of the holotype braincase (IWCMS 2014.95.1–3) and the referred and mirrored postorbital (IWCMS 2014.95.4) shows that the orbits appear more anteriorly facing compared to some other theropods (e.g. *Ceratosaurus*), which display the plesiomorphic, laterally facing condition (Zelenitsky et al., 2009). If correct, this could indicate a higher degree of stereoscopic vision, which perhaps aided in to the calculation of prey position during hunting. However, we note that stereopsis can evolve in animals with laterally facing orbits, and that it may not necessarily be present in animals with binocular overlap (Nityananda & Read, 2017).

#### 4.1.2 Hearing

The endosseous cochlear duct (housing the cochlea or basilar papilla), is closely associated with hearing performance (sensitivity and frequency range) (Choiniere et al., 2021; Walsh et al., 2009). Indeed, longer cochlear ducts provide improved sensitivity to low‐frequency sounds (Gleich et al., 2005; Walsh et al., 2009). The auditory capabilities of the Wealden Supergroup baryonychines, as well as various other saurischians, are presented in Table 4.

**TABLE 4:** Auditory capabilities of various saurischians based on the equations of Walsh et al. (2009).

Taxon	Specimen number	Cochlear duct length (ECD; mm)	Basicranial length (BCL; mm)	Best hearing frequency (Hz)	Mean hearing frequency (Hz)
*Baryonyx*	NHMUK PV R9951	19.6	104.5	2538	1594
*Ceratosuchops*	IWCMS 2014.95.3	17.7	106.8	2210	1416
*Irritator*	SMNS 58022	18.1	75.3	3196	1950
*Viavenator*	MAU Pv LI 530	15.5	99.1	2057	1333
*Sinraptor*	IVPP 10600	17.8	102.7	2329	1480
*Murusraptor*	MCF PVPH 411	19.2	75.3	3352	2036
*Erlikosaurus*	IGM 100.111	11.1	55.2	2723	1694
*Velociraptor*	IGM 100/976	11.15	34.71	3965	2368
	IGM 100/0977	7.44	24.66	3798	2278
*Alioramus*	IGM 100/1844	17.88	104.63	2292	1460
*Citipati*	IGM 100/3006	8.75	21.42	4602	2713
*Byronosaurus*	IGM 100/0983	7.97	19.24	4639	2733
*Troodontidae indet*	IGM 100/3500	6.68	15.54	4737	2787
*Thecodontosaurus*	YPM 2192	9.3	40.28	3089	1893
*Phuwiangosaurus*	SM K11‐006	9.48	n/a	1687	1132

*Note*: See methods for data collected for *Baryonyx*, *Ceratosuchops*, *Viavenator*, *Sinraptor*, *Murusraptor* and *Erlikosaurus*. Data for *Irritator* from Schade et al. (2020); *Phuwiangosaurus* from (Kaikaew et al., 2023); *Thecodontosaurus* from Ballell et al. (2020); and *Velociraptor* (IGM 100/976) from King et al. (2020). All other data from Hanson et al. (2021).

Archosaurs display elongate cochlear ducts relative to most other reptiles (Hanson et al., 2021). An increased degree of cochlear ducts elongation is observed in several theropods and is potentially related to adaptations for auditory foraging (Choiniere et al., 2021). Moderate elongation is present in coelurosaurs including hypercarnivorous tyrannosaurids, dromaeosaurids, alvarezsaurids and troodontids as well as secondarily herbivorous therizinosaurs. However, some hypercarnivorous non‐coelurosaurian theropods (e.g. *Viavenator* and *Sinraptor*) and some herbivorous/omnivorous coelurosaurs (e.g. oviraptorids and ornithomimosaurs) have relatively short ducts, suggesting that they relied on other senses (Choiniere et al., 2021).

Although some works have critiqued the use of Walsh et al.'s (2009) equations (Knoll et al., 2021), they have been commonly used as an approximation of hearing capabilities in extinct taxa and provide a basis for comparisons (Table 4). Previous works estimated that most saurischians have an optimal hearing frequency between 3500–5500 Hz (akin to modern birds) and best mean hearing frequencies between 2250–3250 Hz (Hanson et al., 2021), although the extinct sample was biased towards smaller maniraptoran taxa. However, Gleich et al. (2005), using a different method, suggested that large dinosaurs were capable of low‐frequency perception up to 3000 Hz. The baryonychines share gross hearing capabilities in common with other large theropods such as abelisaurids, allosauroids and tyrannosauroids in having lower‐pitched optimal frequency ranges (Table 4). *Baryonyx* and *Ceratosuchops* also both possessed lower estimated auditory capabilities relative to those reported for *Irritator* (Schade et al., 2020), suggesting differing hearing ecologies between the clades.

The lack of extensive tympanic pneumaticity in either baryonychine also suggests that, like *Irritator* (Schade et al., 2020), the middle ear system was not as specialised for the reception of low‐frequency sounds as those of tyrannosaurids (Witmer & Ridgely, 2009). Extensive pneumaticity (and thus volume of these sinuses) in this region affects the impedance‐matching capabilities of the middle ear and permits the emphasis of low‐frequency sounds (Witmer and Ridgely (2009), and references therein), which spinosaurids were seemingly less reliant upon.

#### 4.1.3 Equilibrium and head posture

Semicircular canal shape has been previously used to infer aspects of spinosaurid ecology: Schade et al. (2020) discussed the possible behavioural ecology of *Irritator* based on the elongate anterior semicircular canal, which they considered to impart enhanced sensitivity to pitch‐down movements of the head. The morphology of the vestibular labyrinth observed in both *Irritator* and the Wealden Supergroup baryonychines (i.e. vertically tall anterior semicircular canals) meanwhile resembles the bipedal locomotor morphotype described in Hanson et al. (2021). While the bipedal nature of baryonychines is now uncontroversial, it is of historic interest that *Baryonyx* was initially considered to be quadrupedal (Charig & Milner, 1986), a stance that was revised in subsequent works (Charig & Milner, 1997). However, it is becoming increasingly difficult to support correlations between semicircular canal geometry and specific ecological or locomotor functions, which may instead be linked to spatial and developmental constraints (Benson et al., 2017; Bronzati et al., 2021; David et al., 2022; Evers et al., 2022). We note that some quadrupedal dinosaurs, such as *Spinophorosaurus* (Knoll et al., 2012), also possess vertically tall anterior semicircular canals, as do some pterosaurs such as *Anhanguera* (Witmer et al., 2003), and increases in relative height of the anterior semicircular canal may not only be affected by bipedal locomotion but also erect limb postures (Bronzati et al., 2021). Taken together, the above inferences regarding spinosaurid locomotion/ecology and semicircular canal geometry are incompatible with the current consensus.

Similarly, previous trends that used lateral semicircular canal orientation to infer “alert” or “habitual” head posture have since been questioned (Benoit et al., 2020; Marugán‐Lobón et al., 2013). Nevertheless, independent support for a down‐turned position of the skull in *Irritator* was provided by the slight ventral rotation of the occipital condyle (Schade et al., 2020; Sues et al., 2002). Such rotation is absent in *Baryonyx* and *Ceratosuchops*, with the condyle projecting largely posteriorly in the baryonychines. Thus, regardless of lateral semicircular canal orientation, the craniocervical articulation suggests differing “standard” head postures within Spinosauridae.

#### 4.1.4 Olfaction

*Ceratosuchops* possess raw olfactory ratios (olfactory bulb length: 26.4 mm; cerebral hemisphere diameter: 52.2 mm) similar to those of most other non‐maniraptoran theropods (Table 5). When the log‐transformed olfactory ratio and estimated body mass are considered and subsequently plotted onto the graph in Zelenitsky et al. (2009: Figure 2), the generalised baryonychine olfactory ratio is near that predicted for a theropod of this size. Baryonychines thus possess the “typical” (c.f. plesiomorphic) condition amongst Mesozoic theropods (Zelenitsky et al., 2009). Such olfactory capabilities are shared with ceratosaurs, allosauroids and basal tyrannosauroids, with the taxa concerned perhaps being less reliant on olfaction than tyrannosaurids and dromaeosaurids (Zelenitsky et al., 2009).

**TABLE 5:** Olfactory ratios of select theropods.

Taxon	Olfactory ratio (%)
Spinosauridae	
*Ceratosuchops*	50.6
Ceratosauria	
*Ceratosaurus*	48.1
*Majungasaurus*	48.3
*Carnotaurus* ^a^	50
*Viavenator* ^b^	57
Allosauroidea	
*Allosaurus*	50–51.6
*Acrocanthosaurus*	58.1
*Carcharodontosaurus*	56
*Giganotosaurus*	57.7
*Sinraptor* ^a^	55
Tyrannosauroidea	
*Dilong*	27
*Tarbosaurus*	65.1–67.4
*Tyrannosaurus*	68.3–71
*Murusraptor* ^c^	45–50
Ornithomimosauria	28.2–32.5
Oviraptoridae	31.5
Dromaeosauridae	28.5–36
Troodontidae	32.6–33.5

*Note*: Data from Zelenitsky et al., (2009, 2011), and supplemented by

^a^ Cerroni and Paulina‐Carabajal (2019),

^b^ Paulina‐Carabajal and Filippi (2018) and

^c^ Paulina‐Carabajal and Currie (2017).

Regarding spinosaurine olfactory bulbs, Arden et al. (2019) suggested that the reduced condition of the bulbs based on frontal specimens from the Ifezouan Formation of Morocco was consistent with an “aquatic” lifestyle. The correlation (or lack thereof) between spinosaurid olfactory bulb size and aquatic ecology was commented on by Hone and Holtz Jr (2019). We supplement this discussion with the discovery that baryonychines did not (based on the mass estimates mentioned above) possess reduced olfactory bulbs relative to their body size; our results indicate retention of the ancestral theropod condition in spinosaurids. Comments regarding the spinosaurine condition thus await better‐preserved specimens that allow for more accurate measurements of endocranial features and estimations of mass.

### 4.2 Implications of baryonychine sensory systems

Consensus exists that spinosaurids exhibit specialisation for some (almost certainly varying) degree of aquatic or semiaquatic adaptation. This view has to be evaluated within the broader debate on which endocranial features are indicative of semi‐aquatic or aquatic behaviour amongst archosaurs. Some studies suggest that shape changes in the inner ear of various amniotes correlate with transitions from terrestrial to semi‐ and fully‐aquatic ecologies (Neenan et al., 2017; Schwab et al., 2020; Spoor et al., 2002), for example. Others, as mentioned above, find no evidence for change in labyrinth shape being associated with such transitions and, furthermore, question previous form‐function inferences based on vestibular geometry (Bronzati et al., 2021; Evers et al., 2022). Furthermore, derived ecologies can arise in the absence of significant shifts in endocranial anatomy, as observed in the extant diving bird *Cinclus* (Passeriformes: Cinclidae), whose endocasts and inner ears are very similar to non‐diving passeriform relatives (Smith et al., 2022).

The grossly comparable endocranial morphologies and REQ scores, relative to other non‐coelurosaurian tetanurans and ceratosaurs, suggests that baryonychines shared similar cognitive capacities with terrestrial, hypercarnivorous forms. This might indicate that spinosaurids were pre‐adapted in terms of neural and sensory adaptations for the exploitation of aquatic prey, or that modification of the rostrum and dentition were sufficient for successful prey capture in aquatic settings and that substantial reorganisation of the endocranium was apparently not required. Further, the unremarkable hearing and olfactory capabilities inferred here also indicates that baryonychines did not deviate substantially from the auditory and olfactory capabilities of “typical” non‐coelurosaurian theropods.

The tactile capabilities of the spinosaurid rostrum have also been deemed important in the context of prey detection and capture in some previous works, with neurovascular networks and patterning of the associated external foramina perhaps imparting crocodile‐like sensitivity to prey movement in water (Ibrahim et al., 2014). Tactile sensation of the face and jaws of vertebrates is detected by the trigeminal nerve (CN V) (Schneider et al., 2016; Vermeiren et al., 2020); however, the available endocranial data for spinosaurids suggest, qualitatively at least, that there is little evidence of an emphasised trigeminal nerve system in these theropods, as is consistent with previous observations of other dinosaur endocasts (Porter & Witmer, 2020). Difficulties persist in collecting the relevant quantitative data in theropod dinosaurs, such as trigeminal ganglion volume, a proxy for facial sensation in crocodyliforms and possibly other archosaurs (George & Holliday, 2013; Lessner & Holliday, 2022). Meanwhile, measurements based on neurovascular foramen size alone would be unreliable due to the likely passage of concomitant vasculature (see above). Whilst some tetanurans with clear adaptations for terrestrial predation possessed rostral neurovascular features akin to spinosaurids and suggestive of tactile jaw edges (Barker et al., 2017; Kawabe & Hattori, 2022), there is no a priori reason to assume theropods had enhanced sensitivity around the oral margin (Porter & Witmer, 2020). Nevertheless, spinosaurids may prove to be an exception (Porter & Witmer, 2020), with the degree of neurovascular branching perhaps more developed relative to other tetanurans (Bouabdellah et al., 2022). Further work, perhaps focusing on the branching pattern of canals within the dentary (which may provide the clearest signal for facial tactile sensation) (Lessner, 2021), for instance, is clearly needed before rostral neurovascular specialisations can be inferred for the clade.

Intriguingly, low‐frequency hearing in the tyrannosaurid *Alioramus* has been tentatively associated with the detection of abnormally low juvenile vocalisations, a possible lack of parental care, or an adaptation for the detection of sounds made by large prey (Hanson et al., 2021). We show that using a larger sample of basal tetanurans and ceratosaurs that low‐frequency hearing is not autapomorphic to *Alioramus* and is seen in a range of large predatory theropod taxa (Table 4). It would be extremely speculative to link this with inferred parental behaviour (or the lack of it), and dietary characteristics related to cochlear duct dimensions failed to produce significant signals (Hanson et al., 2021). Nevertheless, low‐frequency hearing in baryonychines as an adaptation for detecting large prey would not corroborate with evidence of spinosaurids as predators of generally small vertebrates (Hone & Holtz Jr, 2017; Therrien et al., 2005), as shown by their relatively weak bite force for instance (Sakamoto, 2022). Indeed, small prey items were likely commonly selected and depredated by carnivorous theropods in general (Hone & Rauhut, 2010).

## 5 CONCLUSIONS

*Baryonyx* and *Ceratosuchops* possess well‐preserved endocranial features, and provide insight into the evolution of the brain and sensory systems in earlier branching spinosaurids. The overall morphology of the reconstructed endocasts is reflective of their phylogenetic position and follows previous observations of endocranial conservatism in non‐maniraptoriform theropods. Similarly, the morphology of the endosseous labyrinth, and in particular the shape of the semicircular canals, is comparable to other non‐coelurosaurian tetanurans. REQ values imply that baryonychines did not deviate substantially in terms of cognitive capacity and behavioural sophistication relative to other basal theropods, and these predators possessed unexceptional hearing and olfactory capabilities on par with several other large‐bodied terrestrial theropods. There is no tangible evidence for adaptations to semi‐aquatic ecologies in the baryonychine endocrania, suggesting that neural and sensory systems of spinosaurids were pre‐adapted for the successful detection and capture of aquatic prey, or that the initial transition to semi‐aquatic ecologies simply required modifications of the craniodental apparatus.

## AUTHOR CONTRIBUTIONS

CTB, NJG, DN, CEC and PS devised the project. CTB wrote the first draft of the manuscript, conducted the analyses and created the figures. KR optimised the scanning of the *Ceratosuchops* braincase. CTB, JT and LVM segmented the *Ceratosuchops* micro‐CT dataset; CTB reconstructed the full endocast and associated neurovasculature. RR and LW reconstructed the *Baryonyx* endocast and wrote the associated segmentation methodology and scanning protocol. All authors edited the manuscript.

## Supporting information


**Data S1.** Supporting Information

## Data Availability

The data that support the findings of this study are available on Morphosource (https://www.morphosource.org/projects/000491312).
