# Supplementary material for: Modified skulls but conservative brains? The palaeoneurology and endocranial anatomy of baryonychine dinosaurs (Theropoda: Spinosauridae)
Source: J Anat. 2023 Feb 13;242(6):1124–45. doi: 10.1111/joa.13837 (PMC10184548; doi:10.1111/joa.13837)
Supplement: Supplementary file 1 — Data S1. Supporting Information [file JOA-242-1124-s001.pdf]

Supplementary Information: Modified skulls but conservative brains? The palaeoneurology and endocranial anatomy of baryonychine dinosaurs (Theropoda: Spinosauridae)

Chris Tijani Barker[1,2], Darren Naish[3], Jacob Trend[3], Lysanne Veerle Michels[3], Lawrence Witmer[4], Ryan Ridgley[4], Katy Rankin[5], Claire E. Clarkin[1], Philipp Schneider[6,7] & Neil J. Gostling[1,3]**

[1] Institute for Life Sciences, University of Southampton, University Road, Southampton, SO17 1BJ, UK
[2] Faculty of Engineering and Physical Sciences, University of Southampton, University Road, Southampton, SO17 1BJ, UK
[3] School of Biological Sciences, Faculty of Environment and Life Sciences, University of Southampton, University Road, Southampton, SO17 1BJ, UK
[4]Department of Biomedical Sciences, Heritage College of Osteopathic Medicine, Ohio Center for Ecology and Evolutionary Studies, Ohio University, Athens, Ohio, 45701 USA
[5] μ-VIS X-ray Imaging Centre, Faculty of Engineering and Physical Sciences, University of Southampton, SO17 1BJ, Southampton, UK
[6]Bioengineering Science Research Group, Faculty of Engineering and Physical Sciences, University of Southampton, Southampton, UK
[7]High-Performance Vision Systems, Center for Vision, Automation and Control, AIT Austrian Institute of Technology, Vienna, Austria

*Corresponding Authors: Neil J. Gostling: N.J.Gostling@soton.ac.uk, (023) 8059 8141; Chris T. Barker: ctb1g14@soton.ac.uk.

Methodology

Here we provide additional information regarding the rearticulation of the *Ceratosuchops* braincase and generation of the endocast figured and described in the main text. An overview of our workflow is provided in Fig. S1.

Braincase rearticulation and slice generation

We used 3D Slicer (v. 4.11.20210226) to rearticulate STLs of the braincase components via the registration of landmarks using the *Fiducial Registration Wizard* module, available through the *IGT extension* (Ungi et al., 2016). The registration of landmarks borrows from the method outlined by Carvalho et al. (2022). Alignment was achieved by the sequential addition of “floating” elements (the isolated right otoccipital of IWCMS 2014.95.3, followed by IWCMS 2014.95.2

and 2014.95.1) to a "static" element (the isolated basioccipital-basisphenoid complex IWCMS 2014.95.3).

Firstly, segmented braincase regions of interest (ROIs) were converted into contour meshes in ORS Dragonfly (*3D Modelling>Generate Contour Mesh*), exported as STLs (*Export>Mesh to File*), and then imported into 3D Slicer. Here, landmark point pairs were inserted, in the same order, on to the "static" element (*Fiducial Registration Wizard>From fiducials>Create new MarkupsFiducial*) and on the equivalent contacts on the "floating" elements (*Fiducial Registration Wizard>To fiducials>Create new MarkupsFiducial*).

A new linear transform node was generated (*Place fiducials using transforms>Registration result (From->To) transform>Create new LinearTransform*), with parameters set to *Rigid*, *Manual* and *Auto-update*. Application of this linear transformation was achieved in the *Data* module using the "static" model (*Subject hierarchy>Node>Applied transform* column). The rearticulated models were merged by first hardening the linear transformation (*Subject hierarchy>Node>Applied transform* column>*Harden transform*), followed by the *Merge Models* function in the *Surface Model* module. The newly rearticulated model now formed the basis of the next landmark registration, acting as the "static" model.

We consider the fully rearticulated braincase generated here to represent a "best fit" reconstruction. Whilst most contacts are relatively "clean", we do note that the left prootic likely possesses a minor degree of taphonomic distortion. This has resulted in the merging of this element with the ipsilateral otoccipital in its dorsal part, and we were unable to achieve the natural overlap of the former over the latter without sacrificing contacts elsewhere. This, however, has not impacted the morphology of the endocranial cavity.

Whilst 3D slicer can convert 3D models to 2D image stacks (e.g. *3D Slicer documentation>Developer Guide>Script repository>Rasterize a model and save it to a series of image files*) necessary for the segmentation of the endocranial cavity, this function repeatedly caused the computer to crash. Instead, we exported the rearticulated braincase STL from 3D Slicer and used *Voxelizer* (available at: https://drububu.com/miscellaneous/voxelizer/?out=obj) to convert the rearticulated braincase to a 2D image stack for segmentation of the endocranial cavity. To reduce the computational needs for this conversion, the rearticulated braincase STL was edited in Meshmixer (Autodesk Inc.) using the *Lasso* tool to remove portions of the braincase that did not immediately delineate the endocranium. This reduced braincase STL was subsequently converted into an image stack containing 1005 slices, the latter being reimported into ORS Dragonfly for segmentation. Unfortunately, this method does mean the rearticulated braincase data lacks appropriate voxel dimensions, and an inability to readjust to a common resolution using median voxel size (i.e. 103.3μm^3; R. Broadman, pers. comms., 2022) meant STLs of the endocranial cavity and CN II-V had to be scaled manually (see below).

Segmentation

Four disarticulated bone units contained neurovascular ROIs for segmentation:

1) The skull roof fragment (IWCMS 2014.95.1: CN II–V, both sides);

2) The supraoccipital-left otoccipital complex (IWCMS 2014.95.2: left CN IX–XII, posterior middle cerebral veins, left supraoccipital+otoccipital and right supraoccipital portions of the endosseous labyrinths).
3) A basicranial complex (IWCMS 2014.95.3: left CN VI and VII, right CN IV–VIII, carotid arteries, prootic portion of the endosseous labyrinth);
4) The isolated right otoccipital (IWCMS 2014.95.3: right CN IX–XII, partial endosseous labyrinth);

Segmentation of the individual neurovascular features posterior to cranial nerve (CN) V was conducted on the raw image files of IWCMS 2014.95.2–3, whilst the image stack of the rearticulated braincase generated above was used to segment the endocranial cavity and CN II-V. ROIs were manually segmented in Dragonfly using the *Full* and *Adaptive Gaussian* brush tools between XY, YZ, and XZ planes. Segmentation of the rearticulated endocranial cavity also involved the *Polygon* tool in 3D view.

The right otoccipital is preserved detached from but in close association with the basioccipital-basisphenoid complex (IWCMS 2014.95.3); both are separated by a thin layer of matrix. These ROIs were segmented in 2D using the *Adaptive Gaussian* brush implemented with the *Multi-slice* function, using both upper and lower range functions across the filtered and original slices. All ROIs were submitted to a final round of processing via a Connected Components analysis (*Connected Components>New Multi-ROI (26 connected)>Analysis*) to remove stray voxels using a volume filter, implemented using the *Statistical Properties* of the *Object Analysis* dialog box. ROIs excluding stray voxels were selected and saved.

Endocast reassembly

In order to maintain the relative positioning between the neurovascular features in each bone unit (1-4, see above), individual neurovascular features within each unit were assimilated into a single ROI in ORS Dragonfly. Contour meshes of the new assimilated ROIs, whose properties are listed in Table 1, were generated and saved as STLs.

Table 1 Properties inputted in the *Dataset Tools* tab that were used to generate the neuroanatomical meshes in ORS Dragonfly prior to exportation as STL files.

Mesh No.	Neurovascular components	Threshold	Sampling	Smoothing (no. of iterations)
1	Endocranial cavity	50	2	10
2	Skull roof (CN II–IV)	50	2	5
3	Basicranial complex (left CN VI and VII, right CN VI–VIII, right endosseous labyrinth [prootic portion])	50	3	10
4	Right otoccipital (right CN IX–XII, right endosseous labyrinth [otoccipital portion])	50	3	10

5	Conjoined supraoccipital and left otoccipital complex (left CN IX– XII, left endosseous labyrinth (supraoccipital and otoccipital portions), right endosseous labyrinth [supraoccipital portion])	50	3	10

The STLs of the various neurovascular components were imported into Meshmixer for colouration and reassembly. Colouration of the STL surfaces occurred prior to reassembly and was achieved using the *Sculpt* function. The rearticulated braincase STL generated above was sagittally sliced using the *Plane Cut* function (*Edit>Plane Cut; Cut Type: Cut (Discard Half)*) along its mid-length to serve as a basis for the reassembly of the endocranial and neurovascular components. The better-preserved right side of the braincase was used for this purpose.

Reassembly utilised the *Transform* tool (*Edit>Transform*), using the *3D Transform Widget* to manually move the various STLs. Firstly, the endocast of the endocranial cavity and the CN II-V nerve trunks were combined and manually scaled using the *Uniform Scaling* tool and inserted into the corresponding cavity in the braincase. Landmarks such as the borders of the olfactory tract, CN II–IV foramina, and dorsal expansion of the endocast were used to ensure scaling was accurate. Scaling was not necessary for the more posterior neurovascular components given that these were generated from scan data on known voxel dimensions, and these were serially appended and combined. Various external foramina and endocranial cavity endocast topography were similarly used to ensure accurate reassembly of these latter components.

In order to reassemble the neurovascular components from the supraoccipital-left otoccipital unit (IWCMS 2019.45.2), a contour mesh of the conjoined bones was generated and exported as an STL. Both files were appended to the main endocast model. These were selected and manually superimposed in tandem onto the rearticulated half of the supraoccipital using the *Transform* function, with the bone model subsequently deleted once in place, thus leaving the neurovascular components appropriately reassembled.

Given the disarticulated nature of the *Ceratosuchops* braincase, we acknowledge that the reassembly outlined here represent an estimated, “best fit” reconstruction of the endocast.

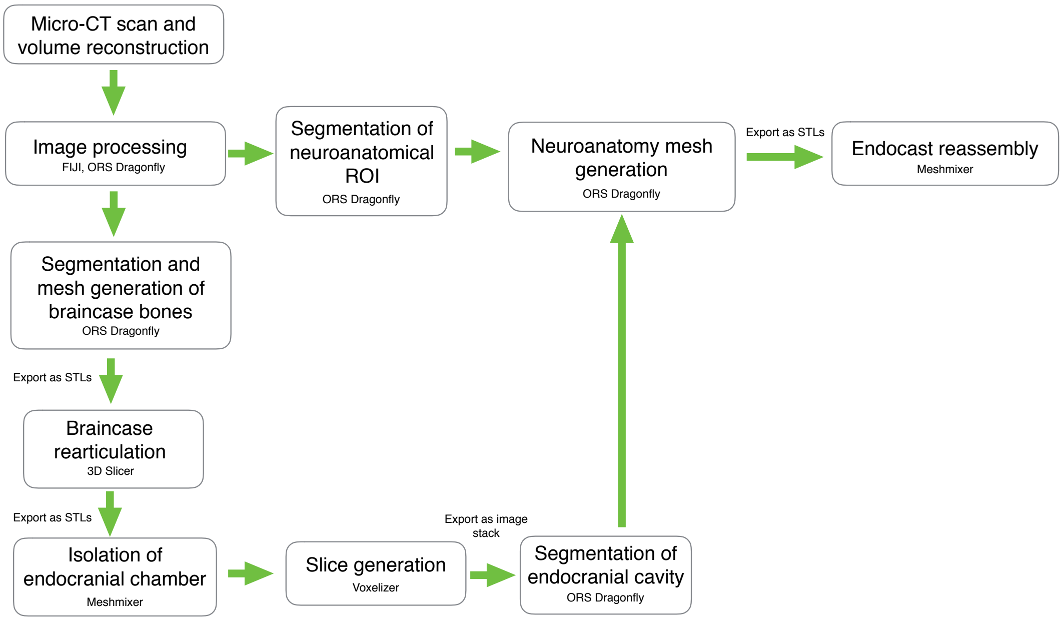

Figure S1. Schematic workflow of the *Ceratosuchops* braincase rearticulation process, segmentation of the endocranial structures, and reassembly of the final endocast described in the main text.

Supplementary References

CARVALHO, F. S. R., DOS SANTOS, T. J. S., NETO, I. C. P., SOARES, E. C. S. & COSTA, F. W. G. 2022. Workflow in open-source software for computed tomography analysis. *British Journal of Oral and Maxillofacial Surgery*.

UNGI, T., LASSO, A. & FICHTINGER, G. 2016. Open-source platforms for navigated image-guided interventions. *Medical image analysis,* 33, 181-186.
